# Stage-Dependent Genetic Association of the TyG Index with Cardiovascular–Kidney–Metabolic Syndrome Severity: A Genome-Wide Association and Mendelian Randomization Study

**DOI:** 10.3390/ijms27146280

**Published:** 2026-07-14

**Authors:** Yu-Lin Ko, Lung-An Hsu, Ngoc Yen Tran, Semon Wu

**Affiliations:** 1 Division of Cardiology, Department of Internal Medicine, Taipei Tzu Chi Hospital, Buddhist Tzu Chi Medical Foundation, New Taipei City 23142, Taiwan; yen.tranngoc41@gmail.com; 2 School of Medicine, Tzu Chi University, Hualien 97004, Taiwan; 3 Department of Research, Taipei Tzu Chi Hospital, Buddhist Tzu Chi Medical Foundation, New Taipei City 23142, Taiwan; 4 Cardiovascular Division, Department of Internal Medicine, Chang Gung Memorial Hospital, Chang Gung University College of Medicine, Taoyuan 33305, Taiwan; hsula@cgmh.org.tw; 5 Department of Life Science, Chinese Culture University, Taipei 11114, Taiwan; semonwu@yahoo.com.tw

**Keywords:** triglyceride–glucose index, insulin resistance, cardiovascular–kidney–metabolic syndrome, mendelian randomization

## Abstract

We aimed to determine whether the genetically predicted triglyceride–glucose (TyG) index, a surrogate marker of insulin resistance, is associated with cardiovascular–kidney–metabolic (CKM) stage severity and whether these associations are consistent with a potential causal role of insulin resistance within the CKM staging framework. The associations between the TyG index and CKM stages were assessed among 107,161 Taiwan Biobank participants using multivariable regression. CKM stages 3 and 4 were combined as the advanced CKM stage group. A genome-wide association study (GWAS) in 104,778 participants identified genetic determinants of the TyG index, followed by Mendelian randomization (MR), weighted genetic risk score (wGRS) analyses, and sample-split MR analyses. CKM stage severity was evaluated using ordinal regression analysis, a sensitivity analysis (CKM stages ≥ 1 vs. stage 0), and a later-stage comparison analysis (CKM stages ≥ 2 vs. stages 0–1). The TyG index increased progressively across CKM stages. GWAS identified 61 genome-wide significant loci associated with the TyG index. MR analyses demonstrated that genetically predicted TyG index was associated with greater CKM stage severity across all models, with consistent findings in the sample-split analyses. Genetic associations were modest for CKM stage 1 versus stage 0 but became substantially stronger for CKM stage 2 and the advanced CKM stages. Genetically predicted TyG index was associated with higher CKM stage severity in a stage-dependent manner, with substantially stronger genetic associations from CKM stage 2 onward. These findings are consistent with a potential causal role of insulin resistance in CKM stage severity and highlight the TyG index as a complementary biomarker for metabolic risk stratification.

## 1. Introduction

The American Heart Association (AHA) recently introduced cardiovascular–kidney–metabolic (CKM) syndrome as an integrated clinical framework that links adiposity, metabolic dysfunction, chronic kidney disease, and cardiovascular disease into a progressive staging system (stages 0–4), providing a comprehensive approach for risk stratification and early prevention [1,2]. This construct emphasizes the shared pathophysiological pathways that precede overt clinical disease, with early to advanced CKM stages representing a critical window for intervention, as even subclinical transitions across these stages are associated with significantly elevated risks for cardiovascular and all-cause mortality [3,4].

Insulin resistance (IR) has been proposed as a central metabolic pathway linking the CKM syndrome continuum. As a key component of metabolic dysregulation, IR links adiposity to systemic inflammation, endothelial dysfunction, and oxidative stress, processes that contribute to atherosclerosis and renal decline [5,6,7]. However, while the association between IR and individual CKM syndrome components is well documented, it remains unclear whether it primarily reflects worsening metabolic dysregulation or contributes to the increasing severity of CKM syndrome.

A major obstacle in large-scale epidemiological research has been the logistical complexity of measuring IR. While the hyperinsulinemic-euglycemic clamp remains the gold standard [8], its invasive nature precludes its use in population-level studies. Several surrogate indices have been developed to estimate IR, including homeostasis model assessment of insulin resistance (HOMA-IR), metabolic score for insulin resistance (METS-IR), and the triglyceride level to high-density lipoprotein cholesterol level (TG/HDL-C) ratio. Among these, the triglyceride-glucose (TyG) index has become one of the most widely used in large population-based studies because it requires only fasting triglyceride and glucose concentrations, correlates well with clamp-measured IR, and does not require insulin measurements [9]. Furthermore, fasting insulin was not routinely measured in all Taiwan Biobank (TWB) participants, whereas fasting glucose and triglyceride concentrations were available for virtually the entire cohort, making the TyG index particularly suitable for the present GWAS and Mendelian randomization (MR) analyses. Although elevated TyG index values have been observationally linked to incident stroke, chronic kidney disease (CKD) and mortality [10,11,12,13], these associations remain susceptible to residual confounding and reverse adiposity–pathology relationships.

MR offers a powerful approach to overcome these limitations by using genetic variants as instrumental variables to investigate potential causal relationships. While MR has suggested links between the TyG index and isolated conditions like fatty liver, hypertension, heart failure, and stroke [14,15,16,17,18], its relationship with the integrated CKM staging framework has not been examined. We hypothesized that the genetically predicted TyG index is associated with higher CKM stage severity across the CKM continuum.

Leveraging the TWB, a large population-based cohort of over 100,000 participants, this study aimed to evaluate the relationship between genetically predicted TyG index and CKM stage severity. Specifically, we investigated whether genetic predisposition to a higher TyG index was associated with higher CKM stages, with stages 3 and 4 combined as the advanced CKM stage group for analysis. This approach may provide insight into the potential role of IR-related metabolic pathways, as reflected by the TyG index, in the CKM staging framework.

## 2. Results

### 2.1. Baseline Clinical Profiles Across CKM Stages

The flow chart of the participant selection process is provided in Supplementary Figure S1. Detailed staging criteria for CKM syndrome are provided in Supplementary Table S1, with the 10-year CVD risk (%) estimated using the PREVENT Score in CKM stage 3 [4,19], (Supplementary Method S2). The distribution of the 107,161 participants across CKM stages was as follows: stage 0 (14.9%), stage 1 (29.5%), stage 2 (52.7%), and advanced CKM stages (stages 3–4) (3.0%). As shown in Table 1, higher CKM stages, particularly stage 2 and above, were associated with progressively less favorable metabolic, cardiovascular, renal, and hepatic profiles. Notably, the prevalence of metabolic syndrome (MetS) was 0.0% in stages 0 and 1 but increased markedly to 40.6% in stage 2 and 61.9% in the advanced CKM stages (*p* < 10^−307^). Higher CKM stages were also associated with incremental increases in body mass index, waist-to-hip ratio, systolic and diastolic blood pressure, and HbA1c, together with progressive declines in estimated glomerular filtration rate (eGFR) and high-density lipoprotein cholesterol (HDL-C) levels.

### 2.2. Increasing TyG Index Across CKM Stages

**Figure 1:**
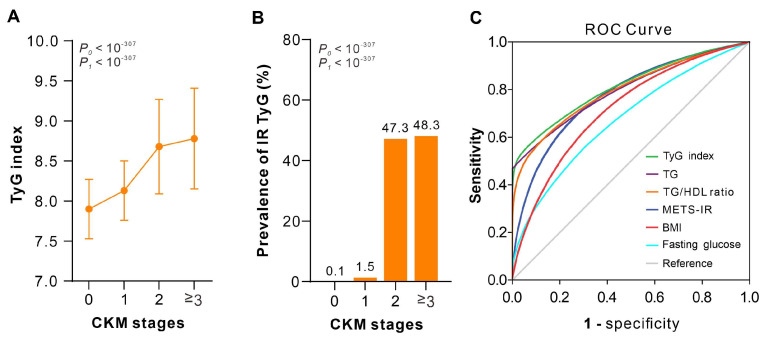
Distribution of TyG index and TyG index-estimated insulin resistance (IR) across cardiovascular–kidney–metabolic (CKM) stages 0–4 with combination of stages 3 and 4 into advanced CKM stages (stages ≥ 3). (**A**) Distribution of TyG index in three CKM stages 0 to advanced CKM stages. (**B**) The prevalence of insulin resistance, defined by the highest quartile of TyG index, across CKM stages 0 to advanced CKM stages. (**C**) ROC curves of the TyG index, METS-IR, BMI, fasting glucose, triglycerides (TG), and the TG/HDL-C ratio for identifying CKM stage 2 and more advanced CKM stages. *P*_0_, unadjusted; *P*_1_, adjusted for age, sex, BMI and current smoking. Abbreviations: CKM, cardiovascular-kidney-metabolic; TyG index, triglyceride–glucose index, ROC curve: Receiver Operating Characteristic, METS-IR, Metabolic Score for Insulin Resistance.

The TyG index increased progressively across CKM stages (*p* < 10^−307^). Observational analyses showed that the prevalence of IR was minimal in CKM stage 0, increased modestly in stage 1, and rose markedly in stage 2 and the advanced CKM stages. Logistic regression further demonstrated a graded association between higher CKM stages and the prevalence of IR (Figure 1). Receiver operating characteristic (ROC) analysis demonstrated that the TyG index had good discriminatory ability for identifying CKM stage 2 and more advanced stages (AUC = 0.8137), outperforming triglyceride alone (AUC = 0.7987), the triglyceride/high-density lipoprotein cholesterol (TG/HDL-C) ratio (AUC = 0.7974), METS-IR (AUC = 0.7766), BMI (AUC = 0.7172), and fasting glucose alone (AUC = 0.6748) (Figure 1C). Using CKM stage 0 as the reference, the odds ratios for IR increased substantially with higher CKM stages across different adjusted models (Supplementary Figure S2). Subgroup analyses demonstrated that these associations were consistent across demographic and lifestyle strata (Supplementary Figure S3).

### 2.3. Genome-Wide Association Study (GWAS) of the TyG Index: Identifying Genetic Determinants

**Figure 2:**
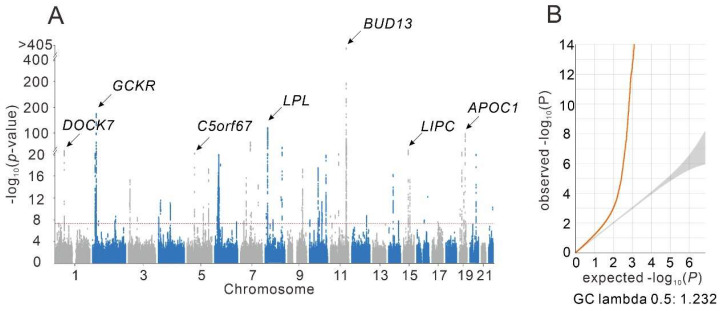
Genome-wide association study of the TyG index. (**A**) Manhattan plot showing genome-wide association results for the TyG index. GWAS analyses were adjusted for age, sex, smoking status, body mass index, and the first 10 principal components to account for population stratification. The red dashed line indicates the genome-wide significance threshold (*p* = 5.0 × 10^−8^). (**B**) Quantile–quantile (QQ) plot of observed versus expected −log_10_(*p*) values, demonstrating appropriate control of population stratification and minimal genomic inflation.

Our GWAS for the TyG index in 104,778 participants identified 61 genome-wide significant loci (Figure 2, Supplementary Table S2). The most significant signal was mapped to the *APOA5* gene cluster on chromosome 11q23.3 (lead single nucleotide polymorphism (SNP) rs7350481 in *BUD13*; *p* < 10^−307^). Other identified loci included key metabolic regulators such as *GCKR*, *LPL*, and *APOC1*, providing a robust set of instrumental variables for subsequent MR analyses.

### 2.4. MR Analysis of the Association Between Genetically Predicted TyG Index and CKM Stage Severity

We constructed a weighted genetic risk score (wGRS) based on the identified genetic variants and performed MR analyses to evaluate the association between genetically predicted TyG index and CKM stage severity (Supplementary Table S3 and Supplementary Figure S4). Excluding participants with a history of hyperlipidemia and/or diabetes mellitus were performed to minimize potential medication effects which showed no substantial alteration of the MR results (Table 2). In the ordinal regression model (CKM stages 0 to advanced CKM stages), genetically predicted TyG index was significantly associated with higher CKM stages (multivariable model, *p* = 1.11 × 10^−88^). In the sensitivity model comparing CKM stages ≥ 1 with stage 0, genetically predicted TyG index was also significantly associated with the presence of CKM syndrome (*p* = 4.21 × 10^−9^).

In the later-stage comparison model comparing CKM stages ≥2 with stages 0–1, genetically predicted TyG index showed an even stronger association with higher CKM stage severity (*p* = 5.12 × 10^−122^) (Table 2). These associations were markedly attenuated after additional adjustment for the measured TyG index, suggesting that the observed genetic association with CKM stage severity was largely mediated through IR-related metabolic pathways, as reflected by the TyG index.

### 2.5. TyG Weighted Genetic Risk Score Demonstrates a Stage-Dependent Association with CKM Stage Severity

**Figure 3:**
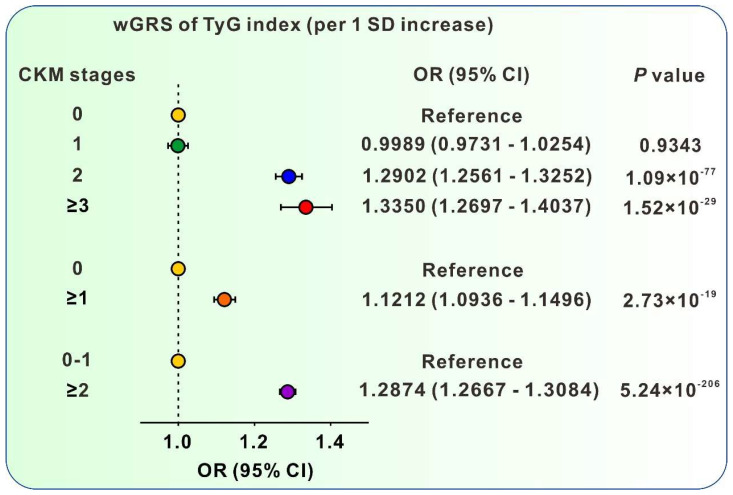
Association of the TyG weighted genetic risk score (wGRS) with CKM stage severity. Forest plot of odds ratios (ORs) and 95% confidence intervals for comparisons between CKM stages per 1-standard deviation increase in the TyG wGRS. A higher TyG wGRS showed little association with CKM stage 1 versus stage 0 but progressively stronger associations with CKM stage 2 versus stage 0, advanced CKM stages (stages 3–4) versus stage 0, and CKM stages ≥ 2 versus CKM stages 0–1. All models were adjusted for age, sex, body mass index, current smoking, and the first 10 principal components. Abbreviations: TyG index, triglyceride–glucose index; wGRS, weighted genetic risk score; CKM, cardiovascular–kidney–metabolic.

To facilitate the clinical interpretation of the genetic findings, we evaluated the association between the TyG weighted genetic risk score (wGRS), constructed from independent genome-wide significant variants, and CKM stage severity. A higher TyG wGRS was associated with progressively greater odds of higher CKM stages. The association with CKM stage 1 versus stage 0 was modest (OR 0.9989, 95% CI 0.9731–1.0254), whereas stronger associations were observed for CKM stage 2 versus stage 0 (OR 1.2902, 95% CI 1.2561–1.3252) and advanced CKM stages versus stage 0 (OR 1.3350, 95% CI 1.2697–1.4037). Moreover, a higher TyG wGRS was significantly associated with CKM stage 2 or above compared with CKM stages 0–1 (OR 1.2874, 95% CI 1.2667–1.3084). Collectively, these findings suggest a threshold-like pattern, with substantially stronger TyG-related genetic associations observed from CKM stage 2 onward (Figure 3).

### 2.6. Sensitivity Analysis

The robustness of the MR estimates was evaluated through multiple sensitivity analyses. Linear regression analyses of the continuous TyG index showed associations in the same direction as the MR estimates, further supporting the robustness of the observed association (Supplementary Table S3). Effect sizes were consistent across inverse variance weighting (IVW), weighted median, and MR-Egger regression (Supplementary Table S4). MR-Egger intercept tests, symmetrical funnel plots (Supplementary Figure S5), and scatter plots (Supplementary Figure S6) indicated an absence of directional pleiotropy. Sensitivity analyses demonstrated significant heterogeneity across instrumental variables (Cochran’s Q and Rücker’s Q tests, *p* < 0.001) (Supplementary Table S5). However, effect estimates remained directionally consistent across IVW, weighted median, and MR-Egger methods, with no evidence of substantial directional pleiotropy, supporting the robustness of the observed associations.

### 2.7. Sample Split MR

In sample-split two-sample MR sensitivity analyses, SNP–exposure (TyG index) associations were estimated in the TWB2 subset (n = 75,402), whereas SNP–outcome (CKM stages) associations were recalculated in the independent, non-overlapping TWB1 subset (n = 20,757). The MR estimates were consistent with the primary analyses across both logistic and ordinal logistic regression models, including analyses excluding participants with a history of hyperlipidemia or with a history of both hyperlipidemia and diabetes mellitus (Supplementary Figure S7, Supplementary Tables S6–S8). These findings suggest that sample overlap did not materially bias the MR estimates.

## 3. Discussion

In this large population-based study, we observed progressively higher TyG index values, a validated surrogate marker of IR, across CKM stages. A major finding was that genetically predicted TyG index showed a stage-dependent association with CKM stage severity, with relatively modest associations for CKM stage 1 and substantially stronger associations for higher CKM stages. ROC analysis demonstrated that the TyG index had the highest discriminatory performance among the evaluated parameters for identifying CKM stage severity. Previous studies have reported associations between the TyG index and individual CKM components or overall CKM staging [9,10,11,12]; however, the relationship between genetically predicted TyG index and stage-specific CKM severity has not previously been evaluated. Observationally, TyG index-estimated IR increased modestly in CKM stage 1 but increased markedly in CKM stage 2 and more advanced stages. Complementary MR analyses further demonstrated that the genetically predicted TyG index was significantly associated with higher CKM stage severity. Together, these findings suggest that the TyG-related genetic association becomes more pronounced from CKM stage 2 onward, exhibiting a stage-dependent, threshold-like pattern of association without establishing a biological threshold.

### 3.1. Stronger IR-Related Metabolic Dysregulation from CKM Stage 2 Onward

A principal finding of this study is the substantially stronger association between IR and CKM stage severity beginning at CKM stage 2. Although CKM stage 1 is characterized by excess or dysfunctional adiposity, individuals at this stage generally remain metabolically compensated, with relatively preserved blood pressure, lipid, and glucose profiles. In the present study, the TyG index increased markedly from CKM stage 2 onward, coinciding with the emergence of overt metabolic abnormalities, including hypertension, hypertriglyceridemia, diabetes, and early chronic kidney disease. These observations are consistent with the adipose tissue expandability hypothesis, whereby adipose tissue buffering capacity eventually becomes insufficient, leading to ectopic lipid deposition, worsening IR, and overt metabolic dysfunction [20,21]. Together, these observations suggest that IR-related metabolic dysregulation becomes substantially more prominent from CKM stage 2 onward and may therefore identify an important stage for earlier preventive intervention.

The TyG index is not intended to replace established diagnostic criteria for metabolic syndrome or CKM syndrome. Rather, it complements conventional anthropometric and clinical measurements by providing a quantitative estimate of IR. Because IR often precedes overt abnormalities in glucose metabolism and may not be fully reflected by body mass index, waist circumference, or blood pressure alone, incorporation of the TyG index may improve the identification of individuals with early metabolic dysfunction. Our MR findings further support its potential utility as a complementary biomarker for metabolic risk stratification within the CKM staging framework. Overall, our findings are consistent with a potential role of IR, as reflected by the TyG index, in higher CKM stage severity. However, because the TyG index shares metabolic components with the CKM staging framework, the observational associations should be interpreted with caution. The MR analyses strengthen the evidence supporting a potential causal role by leveraging genetically predicted TyG and reducing confounding, although they cannot eliminate all potential sources of bias. Accordingly, the TyG index should be interpreted as providing complementary metabolic information rather than duplicating existing CKM diagnostic components.

### 3.2. Clinical and Therapeutic Implications

The genetic associations between the TyG index and CKM syndrome stages have important implications for risk stratification, early detection, and preventive intervention. As a simple, inexpensive, and widely available surrogate marker of IR derived from routinely measured fasting triglyceride and glucose concentrations, the TyG index may serve as a complementary biomarker of IR-related metabolic dysregulation. In our study, the TyG index increased progressively across CKM stages and was strongly associated with higher CKM stages, particularly from the early adiposity-related stage (stage 1) to more advanced metabolic dysfunction. These findings suggest that the TyG index may function as an early metabolic warning marker, enabling the identification of individuals at elevated risk of higher CKM stages before the development of overt cardiovascular disease.

Furthermore, the stage-dependent association observed in the present study suggests that IR-related metabolic dysregulation becomes increasingly prominent from CKM stage 2 onward. Consequently, the TyG index may complement existing CKM risk assessment tools by facilitating earlier identification of individuals at increased likelihood of higher CKM stage severity and supporting more targeted preventive interventions.

From a therapeutic perspective, our findings highlight the potential importance of IR-related pathways in driving CKM stage severity. Although lifestyle modification remains the cornerstone of prevention and management [22], interventions that improve metabolic homeostasis may be particularly relevant during the early stages of CKM syndrome. Pharmacological therapies targeting IR, including metformin and thiazolidinediones [23], as well as agents with established cardiometabolic benefits such as sodium–glucose co-transporter-2 inhibitors and glucagon-like peptide-1 receptor agonists [7,24,25], may warrant further investigation, especially among individuals at the critical transition between stage 1 and stage 2 CKM syndrome. However, the clinical utility of TyG index-guided risk stratification and intervention requires confirmation in prospective and interventional studies.

### 3.3. Strengths and Limitations

The major strength of this study is the large-scale integration of the TWB (n > 95,000), which provided the statistical power necessary for high-resolution GWAS and MR analyses. To reduce potential weak-instrument and sample-overlap bias inherent in one-sample MR analyses, we performed sample-split two-sample MR sensitivity analyses using non-overlapping TWB subsets [26], we sought to reduce potential bias related to sample overlap and to mitigate confounding and reverse causation inherent in observational studies. While previous MR studies have linked the TyG index to individual cardiometabolic conditions, our study extends these findings by evaluating the association between genetically predicted TyG index and CKM stage severity within the integrated CKM staging framework, which encompasses adiposity, metabolic dysfunction, chronic kidney disease, and cardiovascular disease.

However, certain limitations warrant consideration. First, while the TyG index is a validated surrogate, it remains an indirect measure of IR compared with the hyperinsulinemic–euglycemic clamp. Second, because CKM staging incorporates metabolic traits related to triglycerides and glucose, some biological overlap between the TyG index construct and CKM stage components is unavoidable; therefore, the MR findings should be interpreted as reflecting IR-related metabolic pathways, as represented by the TyG index. Third, although sensitivity analyses excluding participants with a history of hyperlipidemia or diabetes yielded consistent results, detailed information on pharmacological treatments affecting triglyceride or glucose levels was not uniformly available, and residual treatment effects cannot be completely excluded. Fourth, our study population was exclusively of Han Chinese descent; given that different ethnicities exhibit varying thresholds for healthy adipose expansion, these findings should be validated in more diverse global cohorts. Fifth, because the present analyses were based on cross-sectional CKM stage classifications, the findings should be interpreted as associations with higher CKM stages rather than direct evidence of longitudinal progression between stages. Although MR analysis strengthens causal inference by using genetically predicted exposure, prospective longitudinal studies are required to confirm temporal transitions across CKM stages. Sixth, although the present findings were internally validated using sample-split MR and multiple sensitivity analyses, external validation in independent cohorts with different ancestral backgrounds is warranted to confirm the generalizability of these findings and to exclude potential population-specific effects. In addition, several limitations relate to the operational definition of CKM staging in this study. Although stages 3 and 4 participants were combined to improve statistical power, these stages represent distinct clinical entities according to the AHA CKM framework. Future studies with larger numbers of Stage 4 participants should evaluate these stages separately. Coronary artery calcium scoring was not available for stage 3 classification, and cardiac biomarkers such as B-type natriuretic peptide (BNP) and cardiac troponins were not included, which may limit the precision of subclinical cardiovascular risk assessment. Furthermore, stage 4 CKM classification relied on self-reported history of cardiovascular disease without systematic clinical adjudication.

## 4. Materials and Methods

### 4.1. TWB Participants

This study used a population-based observational cohort design with one-sample MR and sample-split sensitivity analyses using TWB data. The present study included 129,542 TWB participants with Axiom Genome-Wide CHB 1 or 2 Array genotyping and no prior history of cancer. Data were obtained through questionnaires administered at recruitment centers across Taiwan from 2008 to 2015, with all participants providing written informed consent. The participant selection process is provided in Supplementary Figure S1. The definition of chronic kidney disease, diabetes mellitus, prediabetes mellitus, obesity, central obesity, microalbuminuria, hypertension, hypertriglyceridemia, current smoking, and metabolic syndrome was shown in Supplementary Method S1 [27,28,29]. We initially excluded individuals with third-degree genetic relatedness (identity-by-descent >0.187; n = 7216) or a fasting duration of less than 6 h (n = 4647). This left 117,679 participants for subsequent baseline analysis. From this group, we applied specific exclusion criteria based on the intended analysis. For the TyG index GWAS, we excluded individuals with a self-reported history of hyperlipidemia (n = 8799) or diabetes (n = 4102). For CKM syndrome staging, we excluded individuals with missing CKM-related parameters (n = 304) and those with a self-reported history of heart diseases other than coronary artery disease and stroke (n = 10,214). Following these exclusions, the study population comprised 104,778 and 107,161 participants for TyG index GWAS analysis and CKM staging, respectively. Ultimately, 96,159 participants who met the criteria for both the CKM and GWAS cohorts were included in the MR analysis. The study protocol was approved by the Research Ethics Committee of Taipei Tzu Chi Hospital (approval numbers: 08-XD-005, 31 July 2025) and the Ethics and Governance Council of the TWB (approval numbers: TWBR11107-03, 15 August 2022). All research procedures adhered to the principles outlined in the Declaration of Helsinki.

### 4.2. Staging of CKM Syndrome

CKM syndrome was classified according to the American Heart Association Scientific Statement published by Ndumele et al. [1,2]. The American Heart Association proposed the CKM syndrome, categorized into stages 0 to 4, as a new clinical framework and systemic disease. Detailed staging criteria for CKM stages 0 to 4, with stage 3 and 4 combined as advanced CKM stages, are provided in Supplementary Table S1. Because the number of participants with stage 4 CKM syndrome (established cardiovascular disease, n = 1439) was relatively small, stages 3 and 4 were combined as an “advanced CKM” group for statistical analyses to improve estimation stability and statistical power. This grouping was performed for analytical purposes only and does not imply that the two stages are clinically equivalent. Stage 0 was defined as the absence of CKM syndrome risk factors, characterized by normal renal function, BMI, waist circumference, fasting blood glucose, blood pressure, and lipid profiles, without any self-reported history of CVD. Stage 1 included participants with prediabetes, abdominal obesity and/or dysfunctional adiposity, with the absence of CKD or other metabolic risk factors. Stage 2 encompassed participants with moderate CKD or the presence of metabolic risk factors. Stage 3 comprised participants with a high 10-year CVD risk or severe CKD. The 10-year CVD risk (%) was estimated using the PREVENT Score, which considered the following variable ranges: total cholesterol, HDL-C, systolic blood pressure, and glomerular filtration rate (Supplementary Method S2) [4,19]. Stage 4 comprised participants with a self-reported history of coronary artery disease and stroke.

### 4.3. Definitions of IR According to TyG Index-Estimated IR

The TyG index has been proposed as a surrogate marker for assessing IR due to its practical application, high sensitivity and specificity. The TyG index was calculated as follows: TyG index = ln [Triglyceride level (mg/dL) × fasting plasma glucose level (mg/dL)/2] [9,30]. Participants in the highest quartile of the TyG index were classified as having IR.

### 4.4. Genomic DNA Extraction, Genotyping and GWAS Analysis

Genomic DNA was extracted and genotyped using the Customized Axiom Genome-Wide CHB Arrays (Affymetrix, Santa Clara, CA, USA). After stringent quality control (call rate > 97%, minor allele frequency >0.01, Hardy–Weinberg equilibrium *p* > 1 × 10^−6^), genome-wide imputation was performed using the 1000 Genomes Project Phase 3 (East Asian reference). Post-imputation, 3,640,179 high-quality SNPs were retained. GWAS for the TyG index was conducted using linear regression in PLINK v1.9, adjusting for age, sex, and the first 10 principal components (PCs) to account for population stratification. Genome-wide significance was defined as *p* < 5 × 10^−8^ for GWAS analyses.

### 4.5. MR Analyses

MR analyses were conducted under the assumptions that: (1) selected genetic variants were strongly associated with TyG index (relevance assumption); (2) the variants were not associated with confounders of the TyG index–CKM relationship (independence assumption); and (3) the variants influenced CKM stages primarily through IR-related metabolic pathways, as reflected by the TyG index (exclusion restriction assumption). The primary analysis used a one-sample MR framework within the TWB cohort, supplemented by sample-split sensitivity analyses to minimize bias related to sample overlap. We employed a two-stage least squares (2SLS) MR approach to evaluate the association between genetically predicted TyG index and CKM stage distribution (Supplementary Figure S4). Independent genome-wide significant SNPs associated with TyG index were selected as instrumental variables after quality control and pleiotropy screening. The relevance assumption was supported by the strong association between selected SNPs and TyG index at genome-wide significance. Adjustment for ancestry PCs and exclusion of related individuals were performed to minimize confounding due to population stratification. Genetic variants associated with the TyG index at genome-wide significance (*p* < 5 × 10^−8^) were utilized. To ensure the exclusion of horizontal pleiotropy, we removed lead variants directly associated with CKM stage outcomes (*p* < 0.01) after adjusting for the exposure. A wGRS for the TyG index was constructed by weighting risk alleles using their GWAS-derived beta-coefficients.

In the second stage of 2SLS MR analysis, CKM staging was regressed on the genetically predicted TyG index using ordinal logistic regression to capture the stepwise CKM stage severity (0 to advanced CKM stages) or logistic regression (stages ≥ 2 vs. stages 0–1 and stages ≥ 1 vs. stage 0). Instrument strength was validated using the F-statistic (threshold > 10). To evaluate potential bias arising from sample overlap in the primary one-sample MR design, we conducted a sample-split two-sample MR sensitivity analysis. SNP–exposure (TyG index) associations were estimated in the TWB2 subset (n = 75,402), whereas SNP–outcome (CKM stages) associations were recalculated in the independent, non-overlapping TWB1 subset (n = 20,757). This design ensures independence between exposure and outcome samples and minimizes bias related to sample overlap.

### 4.6. Statistical Analysis and Sensitivity Models

Continuous variables were expressed as mean ± standard deviation, and categorical variables were presented as counts and percentages. Continuous variables were analyzed using linear models and categorical variables were analyzed using logistic or ordinal regression models, with adjustment for age, sex, BMI, and current smoking status. The association between the TyG index and CKM stages was evaluated through three distinct comparison models: Ordinal Regression Model: Linear CKM stage severity across stage 0 to advanced CKM stages; Sensitivity Model: stages ≥ 1 vs. stage 0 (any CKM syndrome burden); and Later-stage comparison Model: stages ≥ 2 vs. stages 0–1 (compromised vs. protected metabolic health). All statistical analyses were performed using IBM SPSS Statistics, version 22 (IBM Corp., Armonk, NY, USA). Participants with missing CKM-related variables were excluded from corresponding analyses. Robustness of MR analysis was assessed using IVW, MR-Egger (to detect directional pleiotropy), and weighted median estimators. Heterogeneity was evaluated via Cochran’s Q and Rücker’s Q statistics. Potential horizontal pleiotropy was minimized by excluding variants directly associated with CKM stages after adjustment for TyG index and by performing MR-Egger analyses.

## 5. Conclusions

Our findings support the TyG index as a complementary biomarker associated with higher CKM stage severity within the CKM staging framework and are consistent with a potential causal role of IR in CKM stage severity. The strongest genetic associations were observed for CKM stage 2 and higher stages, suggesting that IR-related metabolic dysregulation becomes increasingly prominent beyond the earliest CKM stages. These findings further support the potential utility of the TyG index as a complementary biomarker for identifying individuals at increased likelihood of higher CKM stage severity and reinforce the importance of early preventive strategies targeting IR and metabolic dysfunction. Future prospective and interventional studies are needed to determine whether improving insulin sensitivity can modify CKM stage severity or reduce subsequent cardiovascular and renal complications.

## Figures and Tables

**Table 1:** Baseline characteristic of CKM stages 0 to advanced CKM stages in Taiwan Biobank participants.

		Cardiovascular-Kidney-Metabolic Syndrome				
		Non-CKM (Stage 0)	Stage 1	Stage 2	Advanced CKM Stages	*p*
	*N*, %	15,916, 14.9%	31,624, 29.5%	56,435, 52.7%	3186, 3.0%	
Sex	Male	2661, 16.7%	8706, 27.5%	25,563, 45.3%	2284, 71.7%	<10^−307^
Education	Junior high school or under	869, 5.5%	3042, 9.6%	8220, 14.6%	747, 23.5%	8.72 × 10^−24^
	Senior high school	3810, 23.9%	8882, 28.1%	17,375, 30.8%	979, 30.7%	
	College or University	9064, 57.0%	15,759, 49.8%	25,097, 44.5%	1223, 38.4%	
	Graduate studies	2167, 13.6%	3934, 12.4%	5732, 10.2%	236, 7.4%	
Smoking status *	Current smokers	953, 6.0%	2223, 7.0%	6147, 10.9%	693, 21.8%	3.84 × 10^−38^
Drinking status *	Current drinkers	495, 3.1%	1242, 3.9%	4233, 7.5%	313, 9.8%	1.50 × 10^−12^
Physical activity *	Regular exercise	5193, 32.7%	12,106, 38.3%	23,619, 41.9%	1716, 53.9%	1.00 × 10^−6^
CKD *	Positive	0, 0%	0, 0%	1014, 1.8%	570, 17.9%	8.16 × 10^−303^
DM *	Positive	0, 0%	0, 0%	8229, 14.6%	1764, 55.4%	<10^−307^
MetS *	Positive	0, 0%	0, 0%	22,918, 40.6%	1972, 61.9%	<10^−307^
Anthropology	Age (years)	43.47 ± 9.75	47.47 ± 10.55	51.73 ± 10.40	61.27 ± 7.11	<10^−307^
	Waist circumference (cm)	72.10 ± 5.05	82.20 ± 7.99	86.68 ± 10.02	90.72 ± 9.46	<10^−307^
	Waist hip ratio	0.80 ± 0.05	0.85 ± 0.06	0.89 ± 0.06	0.93 ± 0.06	<10^−307^
	Body mass index (kg/m^2^)	20.26 ± 1.43	23.91 ± 2.94	25.45 ± 3.86	26.23 ± 3.66	<10^−307^
Blood pressure status	Systolic BP (mmHg)	104.84 ± 10.12	109.80 ± 9.93	126.22 ± 16.58	134.26 ± 21.99	<10^−307^
	Diastolic BP (mmHg)	64.92 ± 6.96	67.50 ± 6.67	78.16 ± 10.43	78.02 ± 12.03	<10^−307^
Lipid profiles	Total cholesterol (mmol/L) ^#^	4.78 ± 0.84	4.98 ± 0.85	5.20 ± 1.00	4.73 ± 0.99	2.30 × 10^−143^
	HDL-Cholesterol (mmol/L) ^#^	1.63 ± 0.33	1.49 ± 0.32	1.32 ± 0.33	1.19 ± 0.28	<10^−307^
	LDL-Cholesterol (mmol/L) ^#^	2.81 ± 0.72	3.08 ± 0.75	3.15 ± 0.90	2.80 ± 0.85	8.94 × 10^−13^
	Triglyceride (mmol/L) ^#^	0.74 ± 0.27	0.89 ± 0.29	1.62 ± 1.15	1.70 ± 1.52	<10^−307^
Glucose metabolism	Fasting plasma glucose (mmol/L) ^††^	4.83 ± 0.30	5.06 ± 0.38	5.38 ± 1.05	5.90 ± 1.61	<10^−307^
	HbA1c (%) ^††^	5.33 ± 0.22	5.60 ± 0.32	5.80 ± 0.75	6.23 ± 1.03	<10^−307^
IR surrogate markers	TyG index ^#,††^	7.90 ± 0.37	8.13 ± 0.37	8.68 ± 0.59	8.78 ± 0.63	<10^−307^
	TG/HDL-C ratio ^#^	1.11 ± 0.54	1.47 ± 0.67	3.21 ± 3.25	3.63 ± 6.26	<10^−307^
Uric acid	Uric acid (µmol/L)	268.25 ± 60.67	302.75 ± 71.38	339.63 ± 81.49	360.45 ± 86.84	<10^−307^
Renal function	Creatinine (µmol/L)	56.58 ± 12.38	60.11 ± 14.14	66.30 ± 17.68	94.59 ± 89.28	1.12 × 10^−160^
	eGFR (mL/min/1.73 m^2^)	111.19 ± 22.57	106.67 ± 22.65	100.10 ± 22.95	82.50 ± 27.40	4.63 × 10^−39^
Liver function	AST(GOT) (U/L)	22.24 ± 11.58	23.50 ± 10.82	26.43 ± 13.10	27.56 ± 13.61	1.27 × 10^−19^
	ALT(GPT) (U/L)	16.60 ± 15.83	20.74 ± 18.94	27.61 ± 22.63	28.21 ± 21.59	1.62 × 10^−86^
	γGT (U/L)	14.91 ± 13.22	19.09 ± 18.95	29.14 ± 39.98	31.43 ± 38.59	1.38 × 10^−158^

Advanced CKM stages were defined as CKM stages ≥ 3. Continuous variables were analyzed using linear models and categorical variables were analyzed using logistic or ordinal regression models. *p*: adjusted for age, sex, BMI and current smoking status; age: adjusted for sex, BMI and current smoking status; sex: adjusted for age, BMI and current smoking status; BMI: adjusted for age, sex and current smoking status; Current smoking status: adjusted for age, sex and BMI. * Definition of lifestyle habits and disease status shown in Supplementary Method S1. ^#^ Exclusion of participants with a history of hyperlipidemia. ^††^ Exclusion of participants with a history of hyperlipidemia and diabetes mellitus. Abbreviations: CKD, chronic kidney disease; DM, diabetes mellitus; MetS, metabolic syndrome; BP, blood pressure; HDL-C, high-density lipoprotein cholesterol; LDL-C, low-density lipoprotein cholesterol; HbA1C, hemoglobin A1C; TyG index, triglyceride-glucose index; eGFR, estimated glomerular filtration rate; AST, aspartate aminotransferase; ALT, alanine aminotransferase; γGT, gamma-glutamyl transferase.

**Table 2:** Summary of coefficients used for standard Mendelian randomization analysis: TyG index to CKM stages in 96,159 participants of the Taiwan Biobank participants.

T_B_	T_A_-T_B_		G_A_-T_A_		G_A_-T_B_		IV_A_-T_B_			IV_A_-T_B_ + T_A_	
CKM Stages	Beta (SE)	*p* ^‡^	Beta (SE)	*p*	Beta (SE)	*p*	Beta (SE)	*p*	*p* ^§^	Beta (SE)	*p*
Stage 0-advanced CKM stages *	1.7638 (0.0153)	<10^−307^	0.9978 (0.0132)	<10^−307^	1.4200 (0.0548)	4.61 × 10^−148^	1.4229 (0.0549)	4.61 × 10^−148^	1.11 × 10^−88^	−0.1071 (0.0587)	0.0680
Stage 0-advanced CKM stages †	1.7362 (0.0147)	<10^−307^	0.9954 (0.0133)	<10^−307^	1.3774 (0.0539)	4.09 × 10^−144^	1.3802 (0.0540)	4.09 × 10^−144^	1.21 × 10^−86^	−0.1372 (0.0576)	0.0172
Stages ≥ 2 vs. stages 0–1 *	2.2092 (0.0197)	<10^−307^	0.9978 (0.0132)	<10^−307^	1.7594 (0.0615)	3.69 × 10^−180^	1.7629 (0.0616)	3.69 × 10^−180^	5.12 × 10^−122^	−0.0563 (0.0694)	0.4170
Stages ≥ 2 vs. stages 0–1 †	2.2243 (0.0195)	<10^−307^	0.9954 (0.0133)	<10^−307^	1.7347 (0.0608)	3.82 × 10^−179^	1.7381 (0.0609)	3.82 × 10^−179^	3.82 × 10^−122^	−0.0811 (0.0688)	0.2381
Stages ≥ 1 vs. stage 0 *	1.2886 (0.0278)	<10^−307^	0.9978 (0.0132)	<10^−307^	0.7630 (0.0930)	2.32 × 10^−16^	0.7645 (0.0932)	2.32 × 10^−16^	4.21 × 10^−9^	−0.3292 (0.0988)	0.0009
Stages ≥ 1 vs. stage 0 †	1.3122 (0.0275)	<10^−307^	0.9954 (0.0133)	<10^−307^	0.7810 (0.0926)	3.43 × 10^−17^	0.7825 (0.0928)	3.43 × 10^−17^	4.51 × 10^−9^	−0.3324 (0.0985)	0.0007

Exclusion of lead variants when the association with outcome showed *p* < 0.01 after exposure adjustment in each MR analysis. * Exclusion of participants with a history of hyperlipidemia and diabetes mellitus. † Exclusion of participants with a history of hyperlipidemia. *p*: Adjusted for age, sex, smoking, body mass index and 10 principal components. *p* ^‡^: Adjusted for age, sex, smoking, body mass index. *p* ^§^: For multivariate analysis, models marked * were further adjusted for alcohol drinking, physical activity, HDL-C, LDL-C, hypertension, chronic kidney disease, education status; whereas models marked † were further adjusted for alcohol drinking, physical activity, HDL-C, LDL-C, hypertension, diabetes mellitus, chronic kidney disease, education status. **Abbreviations:** IV_A_, instrumental variables for G_A_; T_A_: TyG index; G_A:_ wGRS_TyG index_54SNPs; GWAS, genome-wide association study; Beta: beta-coefficient; SE: standard error; wGRS, weighted genetic risk score.

## Data Availability

Upon request, the corresponding author can provide access to the data presented in this study. The data that support the findings of this study are available from Taiwan BioBank but restrictions apply to the availability of these data, which were used under license for the current study, and so are not publicly available. Data are available from the authors upon reasonable request and with permission of Taiwan Biobank.

## Supplementary Materials

The following supporting information can be downloaded at: https://www.mdpi.com/article/10.3390/ijms27146280/s1.

## Abbreviations

The following abbreviations are used in this manuscript:

2SLS	Two-stage least squares
AUC	Area under the curve
BMI	Body mass index
CKD	Chronic kidney disease
CKM	Cardiovascular–kidney–metabolic
CVD	Cardiovascular disease
DBP	Diastolic blood pressure
DM	Diabetes mellitus
eGFR	Estimated glomerular filtration rate
FPG	Fasting plasma glucose
γΓΤ	Gamma-glutamyl transferase
GWAS	Genome-wide association studies
HbA1c	Hemoglobin a1c
HDL-C	High-density lipoprotein cholesterol
HTG	hypertriglyceridemia
HTN	Hypertension
HWE	Hardy–Weinberg equilibrium
IR	Insulin resistance
IVW	Inverse variance weighting
LDL-C	Low-density lipoprotein cholesterol
MAF	Minor allele frequency
MetS	Metabolic syndrome
METS-IR	Metabolic Score for Insulin Resistance
MR	Mendelian randomization
OR	Odds ratio
PREVENT	Predicting Risk of Cardiovascular Disease EVENTs
QQ	Quantile–quantile
ROC	Receiver operating characteristic
SBP	Systolic blood pressure
SNP	Single nucleotide polymorphism
TWB	Taiwan biobank
TyG index	Triglyceride-glucose index
WC	Waist circumference
wGRS	Weighted genetic risk score
