# Supplementary material for: Stage-Dependent Genetic Association of the TyG Index with Cardiovascular–Kidney–Metabolic Syndrome Severity: A Genome-Wide Association and Mendelian Randomization Study
Source: Int J Mol Sci. 2026 Jul 14;27(14):6280. doi: 10.3390/ijms27146280 (PMC13411448; doi:10.3390/ijms27146280)
Supplement: Supplementary file 1 [file ijms-27-06280-s001.zip › ijms-4376654-supplementary.pdf]

Stage-Dependent Genetic Association of the TyG Index with Cardiovascular–Kidney–Metabolic Syndrome Severity: A Genome-Wide Association and Mendelian Randomization Study

Supplementary Method 1

Definitions of chronic kidney disease, diabetes mellitus, prediabetes mellitus, obesity, central obesity, microalbuminuria, hypertension, hypertriglyceridemia, current smoking, metabolic syndrome and metabolic score for insulin resistance

Chronic kidney disease was classified by estimated glomerular filtration rate (eGFR) levels: normal renal function when eGFR >60 mL/min/1.73 m^2 of body surface area, moderate CKD when 30–60 mL/min/1.73 m^2, and severe CKD when eGFR <30 mL/min/1.73 m^2. **Diabetes mellitus (DM)** was defined as a fasting blood glucose level ≥6.94 mmol/L, HbA1c ≥6.5%, or a self-reported history of diabetes. **Prediabetes mellitus** was defined as a fasting blood glucose level ≥5.55 mmol/L and <6.94 mmol/L, or HbA1c ≥5.7% and <6.5%, without a self-reported history of diabetes. **Obesity** was defined as a BMI ≥23 kg/m^2. **Central obesity** was defined as a waist circumference ≥90 cm in men or ≥80 cm in women. **Microalbuminuria** was defined as a urine albumin level ≥30 mg/L. **Hypertension** was defined as a systolic blood pressure (SBP) ≥130 mmHg, diastolic blood pressure (DBP) ≥80 mmHg, or a self-reported history of hypertension. **Hypertriglyceridemia** was defined as a triglyceride level 7.49 mmol/L. **Current smoking** was defined as regular cigarette smoking lasting >6 months, as self-reported by participants at the time of data collection. **Regular exercisers** were classified if participants answered “yes” to either of the following questions: (1) “Do

you exercise regularly?" (defined as at least 30 minutes of exercise on 3 days per week), or (2) "Have you engaged in any exercise in the past 3 months?" [1]. **Metabolic syndrome** was determined using modified criteria from the National Cholesterol Education Program Adult Treatment Panel III (NCEP ATP III) in the absence of medication history. Participant was diagnosed as having MetS if they had at least three of the following five risk factors: (1) Central obesity: WC ≥90 cm in men or ≥80 cm in women; (2) High blood pressure: SBP ≥130 mmHg and/or DBP ≥80 mmHg or a history of hypertension; (3) Low high-density lipoprotein cholesterol (HDL-C): HDL-C <2.22 mmol/L in men, HDL-C <2.77 mmol/L in women; (4) High triglyceride (TG): TG ≥8.32 mmol/L; (5) High fasting plasma glucose (FPG): FPG ≥5.6 mmol/dL or a self-reported history of DM [2]. Metabolic score for insulin resistance (METS-IR) was calculated as follows: [ln (2 × FPG (mg/dL) + TG (mg/dL)) × BMI (kg/m^2)]/[ln (HDL-C (mg/dL))] [3].

References:

1. Hsu LA, Wu S, Tran NY, Chou HH, Ko YL: Genetic Determinants of Leisure-Time Physical Activity in the Taiwanese Population: A Genome-Wide Association Study. Med Sci Sports Exerc. 2025, 57:2736-2745.

2. Grundy SM, Cleeman JI, Daniels SR, Donato KA, Eckel RH, Franklin BA, et al: Diagnosis and management of the metabolic syndrome: an American Heart Association/National Heart, Lung, and Blood Institute Scientific Statement. Circulation. 2005, 112:2735-2752.

3. Bello-Chavolla O.Y., Almeda-Valdes P., Gómez-Velasco D., Viveros-Ruiz T., Cruz-Bautista I., Romo-Romo A., Sánchez-Lázaro D., Meza-Oviedo D., Vargas-Vazquez A., Campos O.A., et al. METS-IR, a novel score to evaluate insulin sensitivity,

is predictive of visceral adiposity and incident type 2 diabetes. Eur. J. Endocrinol. 2018;178:533–544.

Supplementary Figure S1. Participant selection process for analysis. In this study, participants from the Taiwan Biobank study were screened according to predefined inclusion and exclusion criteria. Participants with missing imputation data or cryptic relatedness (identity-by-descent value > 0.187) were excluded during GWAS quality control. *Absence of data on estimated glomerular filtration rate (n = 4), microalbuminuria (n = 198), metabolic syndrome (n = 84) and obesity (n = 18). Abbreviations: CKM, cardiovascular-kidney-metabolic; CKMS, cardiovascular-kidney-metabolic syndrome; GWAS, genome-wide association study; MR: Mendelian randomization; QC quality control.

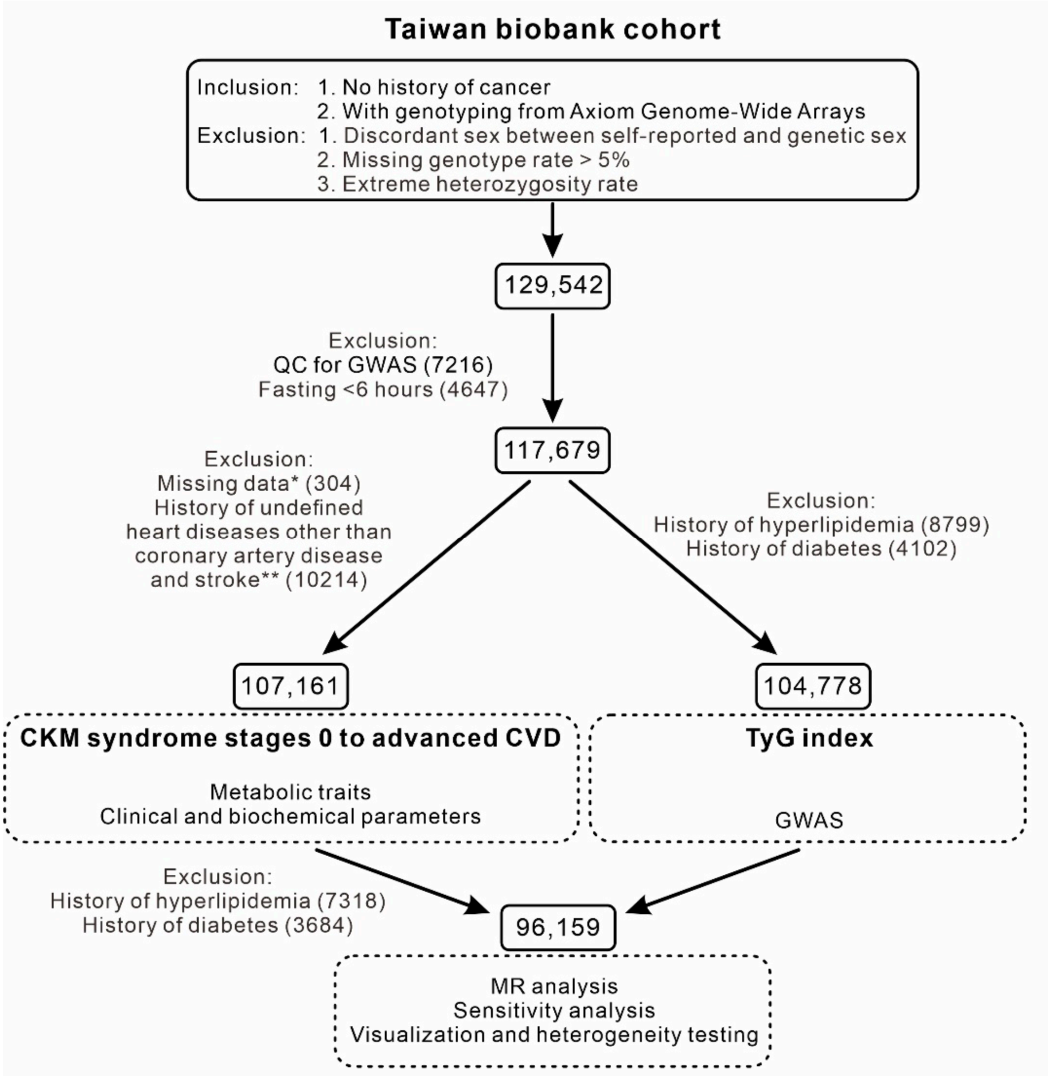

Supplementary Table S1. The classification of CKM stage 0 to advanced stages

CKM stages	CKM staging criteria
Stage 0: No CKM syndrome risk factors	Participants with a normal renal function (eGFR >60 mL/min/1.73 m^2), BMI, waist circumference, blood glucose level, blood pressure, and lipid profile, and no evidence of CKD[a] or subclinical or clinical CVD
Stage 1: Excess and/or dysfunctional adiposity	Participants with prediabetes (fasting blood glucose level: 5.6–6.9 mmol/L), overweight/obesity, abdominal obesity, or dysfunctional adipose tissue (BMI ≥23 kg/m2, waist circumference ≥80 cm for female and ≥90 cm for male, Asian criteria), without the presence of CKD (eGFR >60 mL/min/1.73 m^2) or other metabolic risk factors
Stage 2: Metabolic risk factors or CKD	Participants with moderate CKD (eGFR: 30-60 mL/min/1.73 m^2 or positive for any of albuminuria) or metabolic risk factors, any of the following criteria is met: DM[b], MetS[c], HTN[d] and HTG[e]
Stage 3: Subclinical CVD in CKM syndrome	Participants with high 10-year CVD risk (≥20%) by the PREVENT equation or severe CKD with eGFR <30 mL/min/1.73 m
Stage 4: CVD in CKM syndrome	Participants with a self-reported history of coronary heart disease and/or stroke in combination of one of the metabolic risk factors of CKM syndrome
Advanced Stages of CKM	The combination of Stage 3 and 4

The definition of CKM syndrome is based on the American Heart Association's (AHA) criteria, but there are several key differences to note:

- The cut-off points for BMI and waist circumference in this study follow the AHA-defined Asian criteria.
- The definition of fasting blood glucose (5.6–6.9 mmol/L) and HbA1c (5.7–6.4%) for CKM stage 1 are consistent with the AHA definition.
- The definition of CKD in CKM stages 2–4 is based on the CKD Epidemiology Collaboration (CKD-EPI) formula, which aligns with the AHA definition. However, for albuminuria assessment, the original AHA definition uses albumin-to-creatinine ratio (ACR), whereas this study relies on urine albumin only.

[a]CKD was determined on the basis of eGFR by using the CKD Epidemiology Collaboration (CKD-EPI) formula or by albuminuria defined as a urine albumin level ≥30 mg/L. Normal renal function when eGFR >60 mL/min/1.73 m^2 of body surface area, moderate CKD when 30–60 mL/min/1.73 m^2, and severe CKD when eGFR <30 mL/min/1.73 m^2.

[b]Diabetes (DM) was defined as a fasting blood glucose level ≥125 mg/dL, HbA1c ≥6.5%, or a self-reported history of diabetes.

[c]Metabolic syndrome (MetS) was defined as the presence of at least three of the following five risk factors: (1) Central obesity: WC ≥90 cm in men or ≥80 cm in women; (2) High blood pressure: systolic blood pressure (SBP) ≥130 mmHg and/or diastolic blood pressure (DBP) ≥80 mmHg or a history of hypertension; (3) Low high-density lipoprotein cholesterol (HDL-C): HDL-C <40 mg/dL in men, HDL-C <50 mg/dL in women; (4) High triglyceride (TG): TG ≥150 mg/dL; (5) High fasting plasma glucose (FPG): FPG ≥100 mg/dL or a self-reported history of DM.

[d]Hypertension (HTN) was defined as a SBP ≥130 mmHg, DBP ≥80 mmHg, or a self-reported history of hypertension.

[e]High triglyceride (HTG) levels was defined as a triglyceride level ≥135 mg/dL.

Abbreviation: CKM syndrome: cardiovascular–kidney–metabolic syndrome; BMI: body mass index; CKD: chronic kidney disease; CVD: cardiovascular disease; eGFR: estimated glomerular filtration rate; HbA1c: hemoglobin A1c; WC: waist circumference.

Supplementary Method 2: PREVENT Score Calculation

The American Heart Association (AHA) PREVENT (Predicting Risk of Cardiovascular Disease EVENTs) equations were developed to support the new cardiovascular–kidney–metabolic (CKM) syndrome framework and are recommended for cardiovascular risk stratification within CKM staging systems [1]. The PREVENT equations estimate the 10-year risk of total cardiovascular disease (CVD) in adults aged 30–79 years, with high cardiovascular risk defined as a predicted 10-year CVD risk ≥20%. Because Taiwan Biobank participants were enrolled between 30 and 70 years of age, the PREVENT equations were directly applicable to our study population.

The PREVENT equations were calculated according to the published American Heart Association PREVENT model using sex-specific equations and recommended truncation ranges. The PREVENT model incorporates age, total cholesterol, HDL cholesterol, systolic blood pressure (SBP), estimated glomerular filtration rate (eGFR), smoking status, diabetes mellitus, self-reported history of hypertension, and self-reported history of hyperlipidemia. To maintain consistency with the validated equation ranges, extreme values were truncated as recommended: total cholesterol 130–320 mg/dL, HDL cholesterol 20–100 mg/dL, SBP 90–200 mmHg, and eGFR 14–140 mL/min/1.73 m^2. Values outside these ranges were capped at the corresponding limits (eg, total cholesterol of 330 mg/dL was capped at 320 mg/dL). Subclinical cardiovascular disease assessed by imaging or biomarkers was not included in the current analysis [2].

References:

1. Khan SS, Matsushita K, Sang Y, Ballew SH, Grams ME, Surapaneni A, et al: Development and Validation of the American Heart Association's PREVENT Equations. Circulation. 2024, 149:430-449.

2. Chen Y, Wu X, Long T, Jiang Y, Wang M, Lv Z, et al: Prevalence and Mortality Association of Different Stages of Cardiovascular-Kidney-Metabolic Syndrome. JACC Adv. 2025, 4:101843.

Supplementary Figure S2. Odds ratio of TyG-index-estimated insulin resistance across CKM stages. Forest plots display the odds ratio (OR) and 95% confidence interval (CI) for insulin resistance across cardiovascular–kidney–metabolic (CKM) stages 1, 2, and advanced stages (stages ≥3), using stage 0 as the reference group. Results are shown for three progressively adjusted models: Model 1 (unadjusted), Model 2 (adjusted for sex, age, BMI and current smoking), and Model 3 (adjusted for sex, age, BMI, current smoking, alcohol drinking, physical activity and education). Abbreviations: IR TyG, Insulin resistance derived from triglyceride glucose index; CKM, cardiovascular–kidney–metabolic; OR, odds ratio.

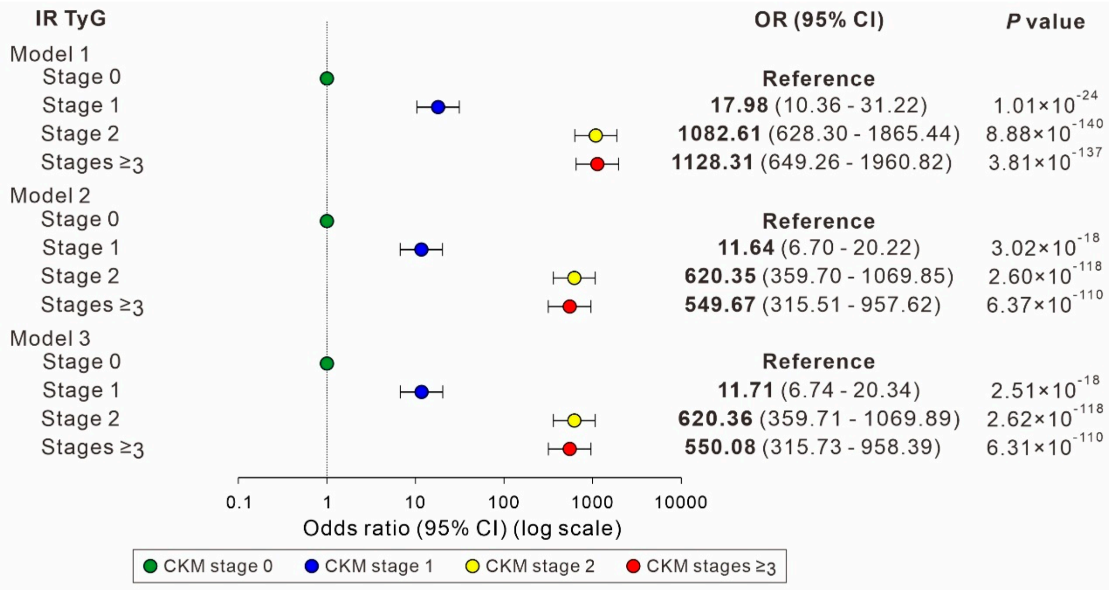

Supplementary Figure S3. Subgroup analysis of odds ratios for TyG-index-estimated insulin resistance across CKM stages (0–advanced). Forest plots show subgroup-specific odds ratio (OR) and 95% confidence interval (CI) for insulin resistance across cardiovascular–kidney–metabolic (CKM) stages 1, 2, and advanced stages, compared with stage 0 as the reference. Subgroups include age (<50, ≥50 years), sex, smoking status, drinking status, exercise habits, and educational level. *P*, adjusted for age, sex, BMI and current smoking. Abbreviations: CKM, cardiovascular–kidney–metabolic; OR, odds ratio.

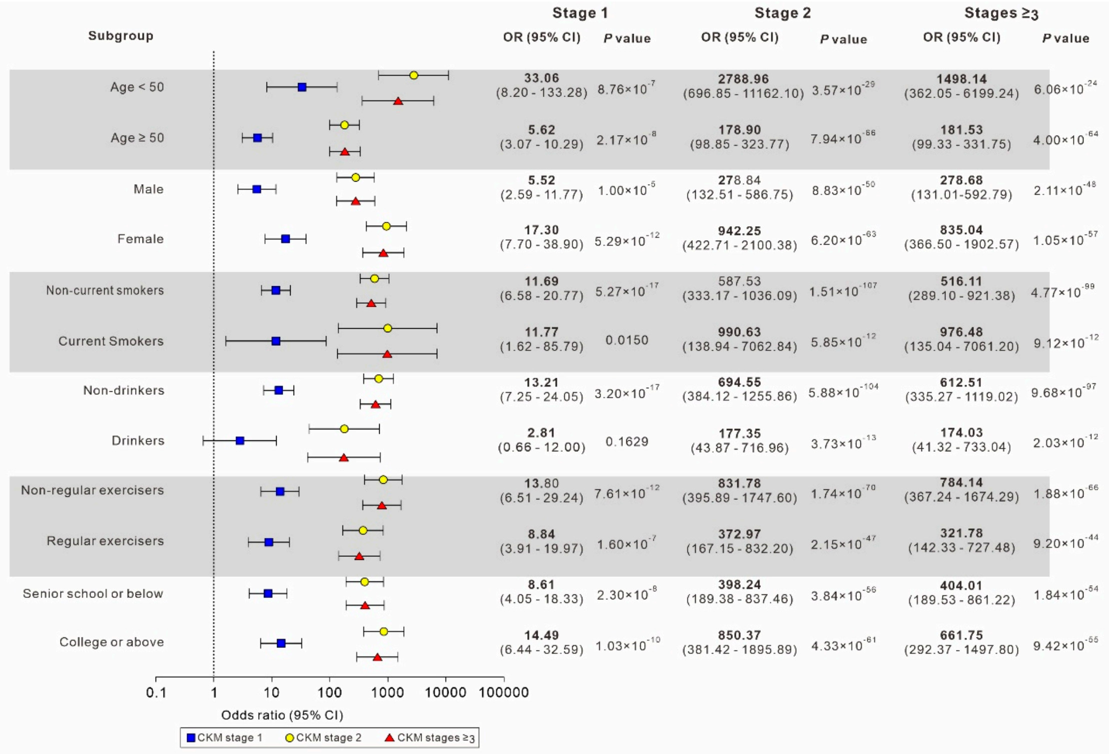

Supplementary Table S2. Lead variants list for GWAS of TyG index and F-statistics for TyG index alleles

CHR	Lead variants	Position (GRCh38)	Gene	Ref / Alt	MAF	HWE	TyG index BETA	SE	L95	U95	*P*	F-statistic	R^2
11	rs7350481	116715567	*BUD13*	C/T	0.2374	0.315	0.1257	0.0026	0.1205	0.1309	$< 10^{-307}$	2309.8678	0.2505
2	rs6547692	27512105	*GCKR*	A/G	0.492	0.3354	0.0656	0.0023	0.0612	0.07	1.16×10^{-184}	2172.0568	0.2401
8	rs6586884	20005847	*LPL*	T/C	0.1011	0.6842	-0.0902	0.0037	-0.0975	-0.0829	1.17×10^{-128}	2162.7279	0.2384
19	rs438811	44913484	*AC011481.3*	C/T	0.1746	0.9292	0.0651	0.003	0.0592	0.0709	1.19×10^{-105}	2148.5485	0.2376
7	rs3812316	73606007	*MLXIPL*	C/G	0.0859	0.8849	-0.0728	0.004	-0.0807	-0.0649	6.15×10^{-73}	2141.6068	0.2366
8	rs2001846	125466208	*AC091114.1*	C/T	0.4369	0.7771	0.0346	0.0023	0.0301	0.0391	8.06×10^{-52}	2131.9236	0.2358
15	rs2070895	58431740	*ALDH1A2, LIPC*	G/A	0.3827	0.463	0.0313	0.0023	0.0268	0.0359	2.34×10^{-41}	2128.4214	0.2355
1	rs11207992	62576129	*DOCK7*	T/G	0.2094	0.1469	-0.0362	0.0028	-0.0416	-0.0307	8.15×10^{-39}	2127.7011	0.2354
19	rs58542926	19268740	*AC138430.1, TM6SF2*	C/T	0.0662	0.6291	-0.054	0.0045	-0.0629	-0.0451	1.10×10^{-32}	2123.8918	0.2352
5	rs465002	56512648	*C5orf67*	T/C	0.4694	0.1535	-0.0253	0.0023	-0.0298	-0.0209	5.30×10^{-29}	2122.0151	0.2352
2	rs56213756	20984123	*AC115619.1*	C/G	0.1209	0.718	0.0384	0.0035	0.0316	0.0452	2.48×10^{-28}	2122.8381	0.235
11	rs174560	61814292	*FADS2, FADS1*	C/T	0.4214	0.0095	-0.0247	0.0023	-0.0292	-0.0201	1.63×10^{-26}	2122.7252	0.235
20	rs148753678	45907567	*PLTP*	CACA/C	0.0668	0.5506	0.0463	0.0046	0.0373	0.0552	3.35×10^{-24}	2093.4524	0.2348
6	rs9264086	31233907	*AL662844.2*	G/A	0.4328	0.2469	0.0229	0.0023	0.0185	0.0274	9.12×10^{-24}	2120.6115	0.2349
10	rs2263985	121143992	*RPL19P16*	T/C	0.4113	0.5373	-0.0216	0.0023	-0.0261	-0.0171	5.73×10^{-21}	2119.5293	0.2351
8	rs1041983	18400285	*NAT2*	C/T	0.4323	0.6938	-0.0211	0.0023	-0.0255	-0.0166	2.71×10^{-20}	2119.2358	0.2348
6	rs1358980	43796814	*AL157371.2*	T/C	0.4486	0.7872	-0.0203	0.0023	-0.0248	-0.0159	4.42×10^{-19}	2120.0956	0.2347
10	rs6479881	63118802	*AL590502.1*	C/T	0.3875	0.6828	0.0204	0.0023	0.0158	0.025	2.16×10^{-18}	2104.3137	0.2348
5	rs6882076	156963286	*TIMD4*	C/T	0.2713	0.2033	-0.0222	0.0025	-0.0271	-0.0172	3.50×10^{-18}	2119.7395	0.2347
9	rs77824033	109478856	*PTPN3*	T/C	0.2296	0.3956	-0.0237	0.0027	-0.029	-0.0184	3.65×10^{-18}	2052.4929	0.2349

14	rs79823890	52045251	*NID2*	G/T	0.1186	0.734	-0.0295	0.0035	-0.0363	-0.0226	3.94×10^{-17}	2118.3794	0.2346
7	rs12534104	44180143	*GCK*	C/A	0.226	0.0646	-0.0227	0.0028	-0.0281	-0.0173	1.55×10^{-16}	2064.6746	0.2356
15	rs77648222	69890512	*DRAIC*	A/G	0.2346	0.6979	-0.0218	0.0027	-0.027	-0.0166	3.47×10^{-16}	2114.7428	0.2347
3	rs7649970	12350773	*PPARG*	C/T	0.0398	0.0577	-0.0469	0.0058	-0.0582	-0.0356	3.74×10^{-16}	2118.8201	0.2346
7	rs34084575	130751643	*KLF14*	T/TA	0.3125	0.1542	-0.0193	0.0025	-0.0241	-0.0145	3.39×10^{-15}	2109.4147	0.2346
6	rs6909732	34251233	*AL354740.1*	C/T	0.1035	0.8781	-0.0288	0.0037	-0.0361	-0.0215	9.06×10^{-15}	2113.3306	0.2347
6	rs198806	26133388	*HIST1H2AC*	G/A	0.1359	0.5134	0.0257	0.0033	0.0192	0.0322	9.26×10^{-15}	2118.3862	0.2346
19	rs1672991	35065755	*HPN, HPN-AS1*	G/A	0.1596	0.4968	-0.0231	0.0031	-0.0292	-0.017	8.25×10^{-14}	2109.3357	0.2347
16	rs2925979	81501185	*CMIP*	C/T	0.4292	0.1979	0.0165	0.0023	0.012	0.021	4.93×10^{-13}	2117.7054	0.2345
8	rs11558471	117173494	*SLC30A8*	A/G	0.4676	0.7192	-0.0164	0.0023	-0.0208	-0.0119	5.60×10^{-13}	2117.6838	0.2345
19	rs141706710	33336121	*RPS3AP50*	C/T	0.159	0.5865	0.0219	0.0031	0.0158	0.028	2.01×10^{-12}	2101.8796	0.2345
4	rs11941723	17810905	*NCAPG*	A/G	0.1433	0.9353	0.0228	0.0032	0.0164	0.0291	2.27×10^{-12}	2111.6157	0.2348
10	rs35978445	92664665	*EIF2S2P3*	A/G	0.3363	0.3303	0.0167	0.0024	0.0119	0.0214	4.71×10^{-12}	2095.504	0.2349
4	rs10023050	87143279	*AFF1*	A/G	0.4101	0.559	-0.0159	0.0023	-0.0204	-0.0113	6.93×10^{-12}	2097.5472	0.2346
5	rs56017758	119404998	*TNFAIP8*	G/A	0.1488	0.651	-0.0211	0.0032	-0.0274	-0.0148	4.53×10^{-11}	2084.8957	0.2347
22	rs7291040	46089093	*MIRLET7BHG*	T/C	0.1186	0.4999	-0.0234	0.0036	-0.0303	-0.0164	4.72×10^{-11}	2065.5813	0.2347
9	rs2575876	104903458	*ABCA1*	G/A	0.2309	0.0246	-0.0175	0.0027	-0.0228	-0.0123	5.09×10^{-11}	2116.914	0.2345
19	rs60748247	8435690	*MARCHF2*	C/CTACCACACCTGGCTAGT	0.0135	0.1339	-0.063	0.0097	-0.0821	-0.0439	1.07×10^{-10}	2114.0414	0.2345
10	rs111567932	72904785	*OIT3*	GT/G	0.2891	0.9669	0.0157	0.0025	0.0108	0.0206	2.88×10^{-10}	2115.4612	0.2344
7	rs531971155	80601582	*CD36*	G/T	0.0199	0.7153	-0.0501	0.008	-0.0659	-0.0344	4.69×10^{-10}	2106.7396	0.2345
8	rs7002551	9320625	*AC022784.1*	T/C	0.0101	0.1962	0.0699	0.0114	0.0477	0.0921	7.35×10^{-10}	2116.0267	0.2344
11	rs11820100	36285791	*COMMD9*	C/T	0.1896	0.4536	-0.0175	0.0029	-0.0232	-0.0118	1.46×10^{-9}	2114.784	0.2344
12	rs117624317	110194076	*IFT81*	C/T	0.0979	0.603	0.0232	0.0039	0.0157	0.0308	1.75×10^{-9}	2073.2597	0.2344
11	rs163177	2817183	*KCNQ1*	T/C	0.4626	0.5676	0.0136	0.0023	0.0091	0.018	2.19×10^{-9}	2116.2742	0.2344

2	rs1402837	168900844	*SPC25*	C/T	0.4042	0.0567	0.0138	0.0023	0.0093	0.0184	2.45×10^{-9}	2116.255	0.2344
12	rs10744776	109255001	*ACACB*	G/A	0.2507	0.1637	-0.0157	0.0026	-0.0208	-0.0105	2.59×10^{-9}	2092.0237	0.2346
6	rs7766070	20686342	*CDKAL1*	C/A	0.3479	0.3975	0.0141	0.0024	0.0095	0.0188	2.96×10^{-9}	2112.9009	0.2344
4	rs2748447	3336104	*RGS12*	A/G	0.3517	0.0793	-0.0141	0.0024	-0.0188	-0.0094	3.16×10^{-9}	2088.0773	0.2341
3	rs73091851	66716141	*AC098969.2*	A/G	0.1971	0.8847	0.0169	0.0029	0.0112	0.0225	4.10×10^{-9}	2076.5646	0.2344
8	rs112784971	58493931	*CYP7A1*	C/T	0.2304	0.8087	-0.0155	0.0027	-0.0207	-0.0102	1.06×10^{-8}	2087.2014	0.2344
10	rs75413156	5221531	*AKR1C4*	C/A	0.1006	0.7531	-0.0215	0.0038	-0.0289	-0.0142	1.09×10^{-8}	2112.232	0.2345
16	rs12149545	56959249	*CETP*	G/A	0.1611	0.8582	-0.0176	0.0031	-0.0236	-0.0115	1.23×10^{-8}	2116.1829	0.2344
2	rs10187501	164675944	*COBLL1*	A/G	0.0802	0.2243	-0.0234	0.0041	-0.0315	-0.0153	1.63×10^{-8}	2111.601	0.2343
14	rs10147971	91999767	*TRIP11*	C/T	0.3247	0.1223	-0.0136	0.0024	-0.0183	-0.0089	1.79×10^{-8}	2105.9886	0.2342
2	rs340515	44961231	*AC012354.1*	T/G	0.4369	0.5939	0.0129	0.0023	0.0084	0.0174	1.88×10^{-8}	2097.3462	0.234
19	rs10403723	45688570	*SNRPD2*	C/T	0.2331	0.8168	-0.0151	0.0027	-0.0204	-0.0099	1.91×10^{-8}	2089.158	0.234
7	rs7798823	26004532	*MIR148A*	C/T	0.4649	0.7266	0.0128	0.0023	0.0083	0.0173	1.97×10^{-8}	2103.8599	0.2346
17	rs549913492	44528392	*GPATCH8*	T/C	0.0103	0.0238	0.0622	0.0111	0.0404	0.084	2.35×10^{-8}	2099.6776	0.2342
6	rs371311	160296741	*SLC22A2*	A/G	0.2868	0.9778	-0.0139	0.0025	-0.0188	-0.009	2.93×10^{-8}	2109.0786	0.2343
1	rs10917386	23472508	*ASAP3*	T/C	0.4165	0.1367	-0.0126	0.0023	-0.0171	-0.0081	3.91×10^{-8}	2109.2055	0.2346
11	rs6486122	13339977	*ARNTL*	C/T	0.3675	0.7412	0.0129	0.0024	0.0083	0.0175	4.50×10^{-8}	2111.1121	0.2342

CHR: chromosome; Ref/Alt: reference/alternate alleles; MAF: minor allele frequency; HWE: Hardy–Weinberg equilibrium; L95: Lower 95% CI, U95%: Upper 95% CI;

TyG: triglyceride glucose

P: adjusted for age, sex, BMI, current smoking and 10 principal components

Supplementary Table S3. TyG index and CKM stages according to TyG index -determining genotypes with genome-wide significance ($P < 5 \times 10^{-8}$) in Taiwan Biobank participants.

CHR	Lead variants	Position (GRCh38)	Gene	Ref / Alt	MAF	HWE	TyG_index			CKMS_stage 0 to advanced stages			CKMS_stage 0 to advanced stages*		
							BETA	SE	*P*	BETA	SE	*P*	BETA	SE	*P*
11	rs7350481	116715567	*BUD13*	C/T	0.2374	0.3150	0.1257	0.0026	$< 10^{-307}$	0.1742	0.0105	2.93×10^{-61}	-0.0171	0.0117	0.1427
2	rs6547692	27512105	*GCKR*	A/G	0.4920	0.3354	0.0656	0.0023	1.16×10^{-184}	0.1055	0.0090	5.51×10^{-32}	0.0129	0.0098	0.1881
8	rs6586884	20005847	*LPL*	T/C	0.1011	0.6842	-0.0902	0.0037	1.17×10^{-128}	-0.1143	0.0148	9.93×10^{-15}	0.0338	0.0161	0.0357
19	rs438811	44913484	*AC011481.3*	C/T	0.1746	0.9292	0.0651	0.0030	1.19×10^{-105}	0.0510	0.0118	1.50×10^{-5}	-0.0311	0.0129	0.0158
7	rs3812316	73606007	*MLXIPL*	C/G	0.0859	0.8849	-0.0728	0.0040	6.15×10^{-73}	-0.1157	0.0159	3.64×10^{-13}	-0.0083	0.0173	0.6303
8	rs2001846	125466208	*AC091114.1*	C/T	0.4369	0.7771	0.0346	0.0023	8.06×10^{-52}	0.0479	0.0090	1.02×10^{-7}	-0.0051	0.0098	0.6015
15	rs2070895	58431740	*ALDH1A2, LIPC*	G/A	0.3827	0.4630	0.0313	0.0023	2.34×10^{-41}	0.0309	0.0092	7.59×10^{-4}	-0.0208	0.0100	0.0380
1	rs11207992	62576129	*DOCK7*	T/G	0.2094	0.1469	-0.0362	0.0028	8.15×10^{-39}	-0.0323	0.0110	0.0032	0.0168	0.0119	0.1584
19	rs58542926	19268740	*AC138430.1, TM6SF2*	C/T	0.0662	0.6291	-0.0540	0.0045	1.10×10^{-32}	-0.0732	0.0179	4.20×10^{-5}	0.0117	0.0194	0.5470
5	rs465002	56512648	*C5orf67*	T/C	0.4694	0.1535	-0.0253	0.0023	5.30×10^{-29}	-0.0615	0.0089	6.12×10^{-12}	-0.0150	0.0098	0.1252
2	rs56213756	20984123	*AC115619.1*	C/G	0.1209	0.7180	0.0384	0.0035	2.48×10^{-28}	0.0515	0.0137	1.72×10^{-4}	-0.0067	0.0150	0.6561
11	rs174560	61814292	*FADS2, FADS1*	C/T	0.4214	0.0095	-0.0247	0.0023	1.63×10^{-26}	-0.0246	0.0091	0.0070	0.0134	0.0099	0.1792
20	rs148753678	45907567	*PLTP*	CACA/C	0.0668	0.5506	0.0463	0.0046	3.35×10^{-24}	0.0132	0.0180	0.4624	-0.0665	0.0196	6.95×10^{-4}
6	rs9264086	31233907	*AL662844.2*	G/A	0.4328	0.2469	0.0229	0.0023	9.12×10^{-24}	0.0218	0.0090	0.0156	-0.0144	0.0098	0.1413
10	rs2263985	121143992	*RPL19P16*	T/C	0.4113	0.5373	-0.0216	0.0023	5.73×10^{-21}	-0.0850	0.0091	7.04×10^{-21}	-0.0485	0.0099	9.18×10^{-7}
8	rs1041983	18400285	*NAT2*	C/T	0.4323	0.6938	-0.0211	0.0023	2.71×10^{-20}	-0.0240	0.0090	0.0077	0.0146	0.0098	0.1364
6	rs1358980	43796814	*AL157371.2*	T/C	0.4486	0.7872	-0.0203	0.0023	4.42×10^{-19}	-0.0362	0.0090	5.40×10^{-5}	-0.0005	0.0098	0.9591
10	rs6479881	63118802	*AL590502.1*	C/T	0.3875	0.6828	0.0204	0.0023	2.16×10^{-18}	0.0335	0.0092	2.78×10^{-4}	0.0005	0.0100	0.9584
5	rs6882076	156963286	*TIMD4*	C/T	0.2713	0.2033	-0.0222	0.0025	3.50×10^{-18}	-0.0272	0.0101	0.0068	0.0072	0.0110	0.5112

9	rs77824033	109478856	*PTPN3*	T/C	0.2296	0.3956	-0.0237	0.0027	3.65×10^{-18}	-0.0587	0.0108	4.81×10^{-8}	-0.0249	0.0117	0.0337
14	rs79823890	52045251	*NID2*	G/T	0.1186	0.7340	-0.0295	0.0035	3.94×10^{-17}	-0.0929	0.0138	1.86×10^{-11}	-0.0504	0.0151	8.09×10^{-4}
7	rs12534104	44180143	*GCK*	C/A	0.2260	0.0646	-0.0227	0.0028	1.55×10^{-16}	-0.0564	0.0108	2.03×10^{-7}	-0.0310	0.0118	0.0088
15	rs77648222	69890512	*DRAIC*	A/G	0.2346	0.6979	-0.0218	0.0027	3.47×10^{-16}	-0.0561	0.0105	1.03×10^{-7}	-0.0201	0.0115	0.0805
3	rs7649970	12350773	*PPARG*	C/T	0.0398	0.0577	-0.0469	0.0058	3.74×10^{-16}	-0.1054	0.0227	4.00×10^{-6}	-0.0150	0.0246	0.5424
7	rs34084575	130751643	*KLF14*	T/TA	0.3125	0.1542	-0.0193	0.0025	3.39×10^{-15}	-0.0716	0.0097	1.36×10^{-13}	-0.0334	0.0106	0.0015
6	rs6909732	34251233	*AL354740.1*	C/T	0.1035	0.8781	-0.0288	0.0037	9.06×10^{-15}	-0.0697	0.0147	2.00×10^{-6}	-0.0254	0.0160	0.1118
6	rs198806	26133388	*HIST1H2AC*	G/A	0.1359	0.5134	0.0257	0.0033	9.26×10^{-15}	0.0548	0.0131	2.70×10^{-5}	0.0240	0.0143	0.0928
19	rs1672991	35065755	*HPN, HPN-AS1*	G/A	0.1596	0.4968	-0.0231	0.0031	8.25×10^{-14}	-0.0161	0.0122	0.1866	0.0207	0.0133	0.1204
16	rs2925979	81501185	*CMIP*	C/T	0.4292	0.1979	0.0165	0.0023	4.93×10^{-13}	0.0388	0.0090	1.60×10^{-5}	0.0062	0.0098	0.5256
8	rs11558471	117173494	*SLC30A8*	A/G	0.4676	0.7192	-0.0164	0.0023	5.60×10^{-13}	-0.0598	0.0089	2.22×10^{-11}	-0.0235	0.0098	0.0158
19	rs141706710	33336121	*RPS3AP50*	C/T	0.1590	0.5865	0.0219	0.0031	2.01×10^{-12}	0.0348	0.0123	0.0045	-0.0001	0.0134	0.9932
4	rs11941723	17810905	*NCAPG*	A/G	0.1433	0.9353	0.0228	0.0032	2.27×10^{-12}	0.0736	0.0128	9.55×10^{-9}	0.0350	0.0140	0.0126
10	rs35978445	92664665	*EIF2S2P3*	A/G	0.3363	0.3303	0.0167	0.0024	4.71×10^{-12}	0.0439	0.0095	4.00×10^{-6}	0.0089	0.0104	0.3920
4	rs10023050	87143279	*AFF1*	A/G	0.4101	0.5590	-0.0159	0.0023	6.93×10^{-12}	-0.0089	0.0091	0.3303	0.0141	0.0099	0.1554
5	rs56017758	119404998	*TNFAIP8*	G/A	0.1488	0.6510	-0.0211	0.0032	4.53×10^{-11}	-0.0589	0.0126	3.00×10^{-6}	-0.0223	0.0138	0.1055
22	rs7291040	46089093	*MIRLET7BHG*	T/C	0.1186	0.4999	-0.0234	0.0036	4.72×10^{-11}	-0.0325	0.0140	0.0205	0.0113	0.0153	0.4582
9	rs2575876	104903458	*ABCA1*	G/A	0.2309	0.0246	-0.0175	0.0027	5.09×10^{-11}	-0.0273	0.0106	0.0097	-0.0035	0.0115	0.7617
19	rs60748247	8435690	*MARCHF2*	C/CTACCACACCTGGCTAGT	0.0135	0.1339	-0.0630	0.0097	1.07×10^{-10}	-0.1594	0.0382	3.00×10^{-5}	-0.0793	0.0414	0.0556
10	rs111567932	72904785	*OIT3*	GT/G	0.2891	0.9669	0.0157	0.0025	2.88×10^{-10}	-0.0023	0.0098	0.8132	-0.0275	0.0107	0.0105
7	rs531971155	80601582	*CD36*	G/T	0.0199	0.7153	-0.0501	0.0080	4.69×10^{-10}	-0.0350	0.0318	0.2711	0.0689	0.0345	0.0458
8	rs7002551	9320625	*AC022784.1*	T/C	0.0101	0.1962	0.0699	0.0114	7.35×10^{-10}	0.0561	0.0450	0.2133	-0.0452	0.0491	0.3571
11	rs11820100	36285791	*COMMD9*	C/T	0.1896	0.4536	-0.0175	0.0029	1.46×10^{-9}	-0.0387	0.0114	6.78×10^{-4}	-0.0172	0.0124	0.1672
12	rs117624317	110194076	*IFT81*	C/T	0.0979	0.6030	0.0232	0.0039	1.75×10^{-9}	0.0716	0.0152	3.00×10^{-6}	0.0407	0.0167	0.0145
11	rs163177	2817183	*KCNQ1*	T/C	0.4626	0.5676	0.0136	0.0023	2.19×10^{-9}	0.0576	0.0089	1.12×10^{-10}	0.0239	0.0098	0.0143

2	rs1402837	168900844	*SPC25*	C/T	0.4042	0.0567	0.0138	0.0023	2.45×10^{-9}	0.0307	0.0091	7.58×10^{-4}	0.0086	0.0100	0.3884
12	rs10744776	109255001	*ACACB*	G/A	0.2507	0.1637	-0.0157	0.0026	2.59×10^{-9}	-0.0287	0.0104	0.0058	-0.0070	0.0113	0.5344
6	rs7766070	20686342	*CDKAL1*	C/A	0.3479	0.3975	0.0141	0.0024	2.96×10^{-9}	0.0703	0.0094	6.24×10^{-14}	0.0294	0.0102	0.0041
4	rs2748447	3336104	*RGS12*	A/G	0.3517	0.0793	-0.0141	0.0024	3.16×10^{-9}	-0.0208	0.0094	0.0263	-0.0049	0.0102	0.6349
3	rs73091851	66716141	*AC098969.2*	A/G	0.1971	0.8847	0.0169	0.0029	4.10×10^{-9}	0.0162	0.0113	0.1534	-0.0069	0.0123	0.5746
8	rs112784971	58493931	*CYP7A1*	C/T	0.2304	0.8087	-0.0155	0.0027	1.06×10^{-8}	-0.0237	0.0107	0.0262	0.0072	0.0116	0.5339
10	rs75413156	5221531	*AKR1C4*	C/A	0.1006	0.7531	-0.0215	0.0038	1.09×10^{-8}	-0.0309	0.0149	0.0375	0.0056	0.0162	0.7316
16	rs12149545	56959249	*CETP*	G/A	0.1611	0.8582	-0.0176	0.0031	1.23×10^{-8}	-0.0339	0.0121	0.0053	-0.0118	0.0133	0.3754
2	rs10187501	164675944	*COBLL1*	A/G	0.0802	0.2243	-0.0234	0.0041	1.63×10^{-8}	-0.0733	0.0164	7.00×10^{-6}	-0.0300	0.0178	0.0916
14	rs10147971	91999767	*TRIP11*	C/T	0.3247	0.1223	-0.0136	0.0024	1.79×10^{-8}	-0.0325	0.0095	6.38×10^{-4}	-0.0158	0.0104	0.1277
2	rs340515	44961231	*AC012354.1*	T/G	0.4369	0.5939	0.0129	0.0023	1.88×10^{-8}	0.0413	0.0090	5.00×10^{-6}	0.0300	0.0099	0.0023
19	rs10403723	45688570	*SNRPD2*	C/T	0.2331	0.8168	-0.0151	0.0027	1.91×10^{-8}	-0.0363	0.0106	6.28×10^{-4}	-0.0187	0.0116	0.1060
7	rs7798823	26004532	*MIR148A*	C/T	0.4649	0.7266	0.0128	0.0023	1.97×10^{-8}	0.0095	0.0090	0.2913	-0.0117	0.0098	0.2344
17	rs549913492	44528392	*GPATCH8*	T/C	0.0103	0.0238	0.0622	0.0111	2.35×10^{-8}	0.1143	0.0443	0.0098	0.0662	0.0480	0.1684
6	rs371311	160296741	*SLC22A2*	A/G	0.2868	0.9778	-0.0139	0.0025	2.93×10^{-8}	-0.0129	0.0099	0.1904	0.0153	0.0108	0.1552
1	rs10917386	23472508	*ASAP3*	T/C	0.4165	0.1367	-0.0126	0.0023	3.91×10^{-8}	-0.0018	0.0091	0.8425	0.0203	0.0099	0.0403
11	rs6486122	13339977	*ARNTL*	C/T	0.3675	0.7412	0.0129	0.0024	4.50×10^{-8}	0.0114	0.0093	0.2180	-0.0121	0.0101	0.2299

CHR: chromosome; Ref/Alt: reference/alternate alleles; MAF: minor allele frequency; HWE: Hardy–Weinberg equilibrium; TyG: triglyceride glucose; CKM: cardiovascular–kidney–metabolic.

P: adjusted for age, sex, BMI, current smoking and 10 principal components, **P*: adjusted for age, sex, BMI, current smoking, 10 principal components and TyG index

Red-highlighted variants were excluded from the instrumental variable set because they showed evidence of direct association with CKM stages outcomes ($P < 0.01$) independent of the TyG index, to minimize potential horizontal pleiotropy.

Supplementary Figure S4. Diagram of standard Mendelian randomization (MR) approaches between TyG index and CKM stage 0 to advanced stages (stages ≥3), CKM stage 1 to advanced stages *vs.* stage 0 or CKM stage 2 to advanced stages *vs.* stages 0 to 1. Abbreviations: TyG, triglyceride glucose; CKM, cardiovascular–kidney–metabolic; wGRS, weighted genetic risk score.

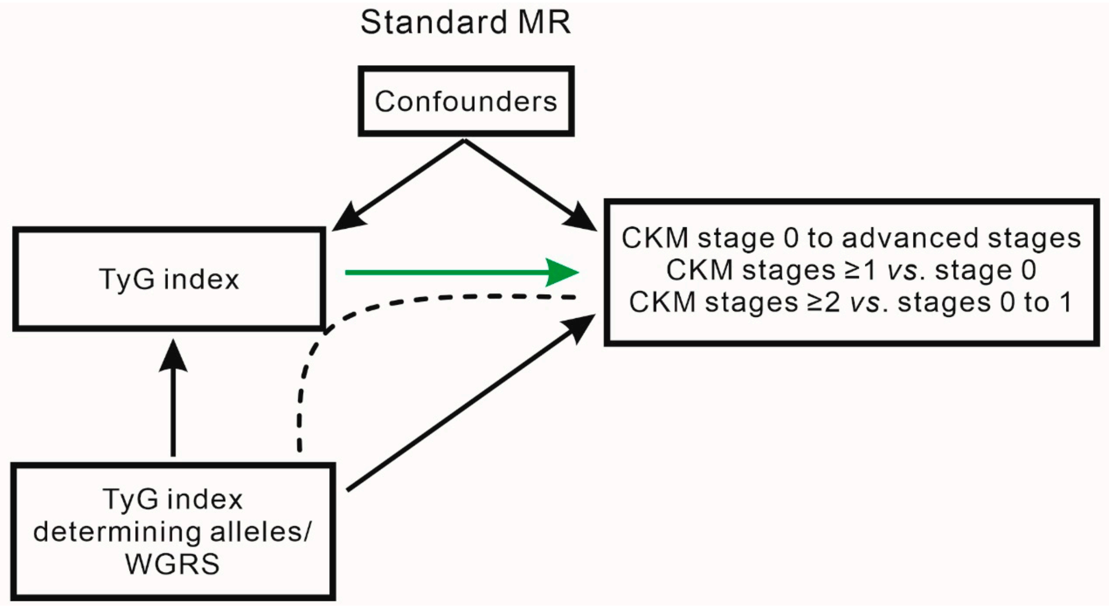

Supplementary Table S4. Sensitivity analyses for the Mendelian randomization association between genetically predicted TyG index and CKM stages

Analysis Method	Coefficient	Standard Error	(95%, CI)	*P* value
	TyG index-determining alleles on CKM stages			
Inverse-variance weighted				
Fixed-effect	1.4759	0.0456	(1.3845, 1.5673)	$< 10^{-307}$
Random-effects	1.6497	0.1115	(1.4261, 1.8733)	$< 10^{-307}$
Egger regression, intercept				
Fixed-effect	-0.0019	0.0025	(-0.0070, 0.0032)	0.4631
Random-effects	-0.0029	0.0030	(-0.0089, 0.0031)	0.3335
Egger regression, slope				
Fixed-effect	1.4766	0.0776	(1.3210, 1.6323)	4.33×10^{-25}
Random-effects	1.4886	0.0803	(1.3274, 1.6498)	1.46×10^{-24}
Simple median	1.5726	0.1446	(1.3702, 1.9284)	< 0.00001
Weighted median	1.3855	0.0179	(1.3855, 1.4185)	< 0.00001

Abbreviations: TyG, triglyceride glucose; CKM, cardiovascular–kidney–metabolic.

Supplementary Figure S5. Funnel plots of the instrumental variable (IV) strength against IV estimates for each genetic variant separately for standard MR for the TyG index on CKM stages. Horizontal lines represent 95% confident intervals for the IV estimates. Solid vertical lines are at the null, and dashed vertical lines are the (fixed-effect) inverse-variance weighted estimates. Instrumental variable strength: $(\frac{\widehat{\beta x}\,|G}{SE\,(\widehat{\beta}Y|G)})$ and instrumental variable estimates: $(\frac{\widehat{\beta Y}\,|G}{\widehat{\beta X}\,|G})$.

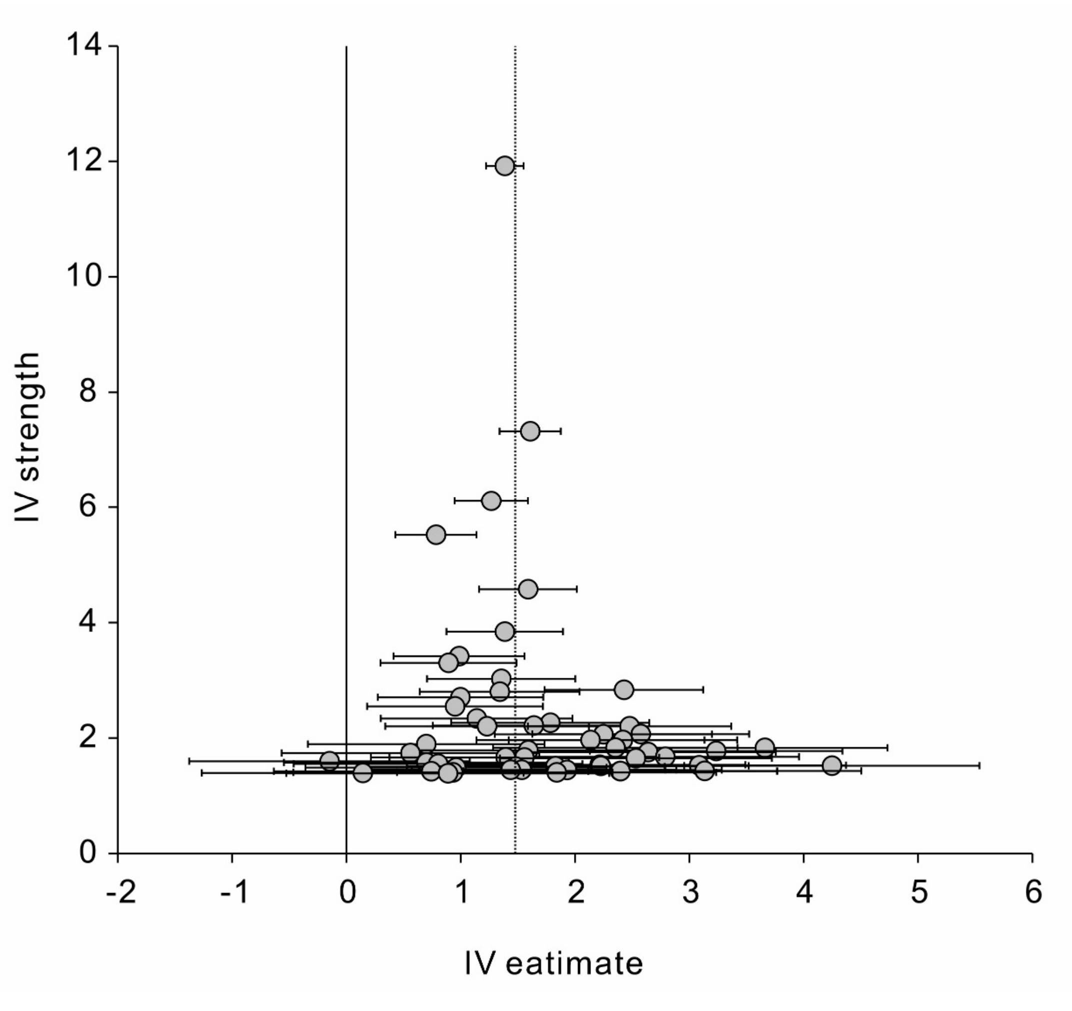

Supplementary Figure S6. Scatter plots showing the associations of TyG index–determining alleles with CKM stages without (A) and with (B) adjustment for TyG index. Each point represents the effect estimate of a TyG index–determining allele on TyG index and CKM stages. Lines represent one standard error (s.e.).

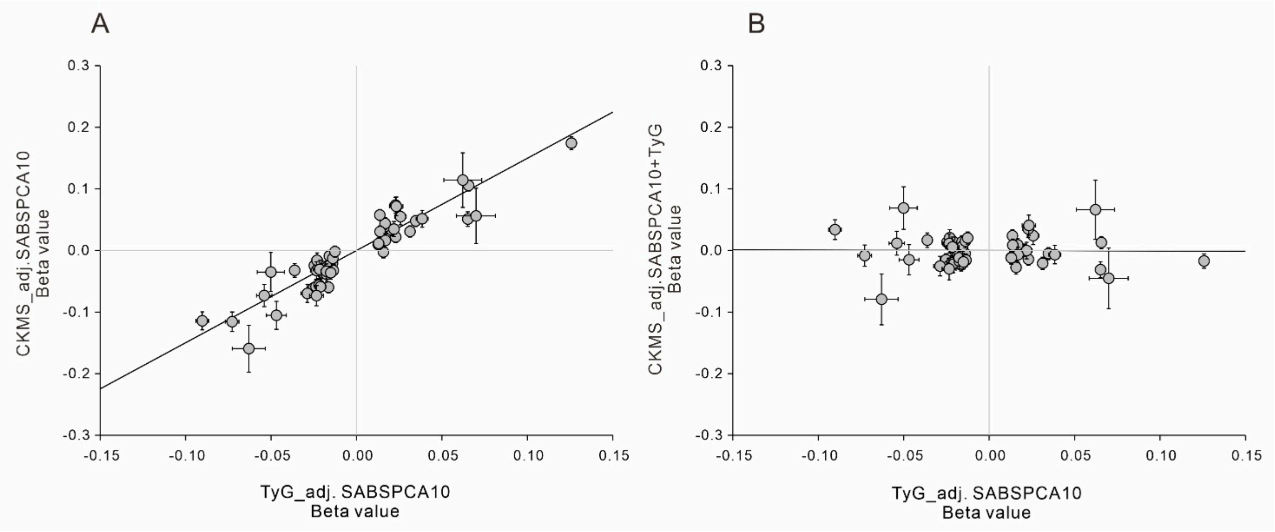

Supplementary Table S5. Cochran's Q and Rücker's Q tests for heterogeneity for MR of TyG index and CKM stages

Exposure[E]	Outcome[O]	Instrumental variables	Analysis Method*	Coefficient*	Standard error	95% confident interval	*P* value	Cochran's Q / Rücker's Q†	P_1 value‡
TyG index	CKM stages	TyG index-determining alleles	IVW method	1.4759	0.0456	(1.3845, 1.5673)	$< 10^{-307}$	152.47	1.46×10^{-11}
			Egger regression, slope	1.4766	0.0776	(1.3210, 1.6323)	4.33×10^{-25}	150.87	<0.0001

I-squared = 65.24% for TyG index -determining alleles

IVW: Inverse-variance weighted

*All with fixed effect

†For IVW method, Cochran's Q test was performed and for Egger regression method, we used Rücker's Q test

‡P_1 value: P value for the Cochran's Q / Rücker's Q tests

Supplementary Figure S7. Genome-wide association study of the TyG index in the TWB2 subset of the Taiwan Biobank. (A) Manhattan plot showing genome-wide association results for the TyG index. GWAS analyses were adjusted for age, sex, smoking status, body mass index, and the first 10 principal components. The red dashed line indicates the genome-wide significance threshold ($P = 5.0 \times 10^{-8}$). (B) Quantile–quantile (QQ) plot of observed versus expected $-\log_{10}(P)$ values.

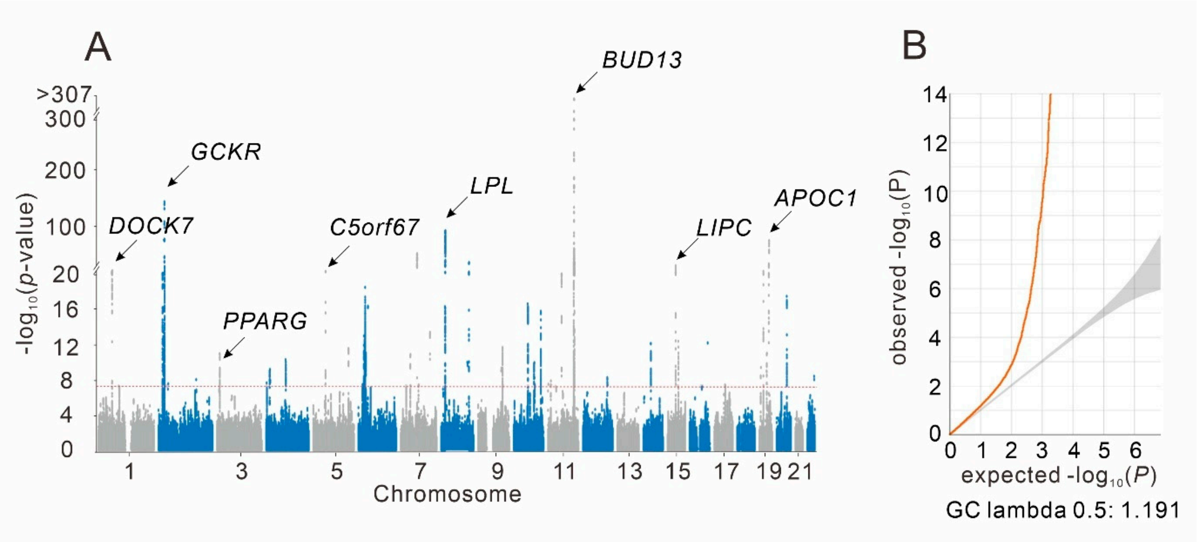

Supplementary Table S6. Lead variants list for GWAS of TyG index for TyG index alleles in TWB2 cohort

CHR	Lead variants	Position (GRCh38)	Gene	Ref/Alt	MAF	HWE	TyG index		
							BETA	SE	*P*
11	rs7350481	116715567	*BUD13*	C / T	0.2374	0.315	0.1248	0.0030	$< 10^{-307}$
2	rs6547692	27512105	*GCKR*	A / G	0.492	0.3354	0.0665	0.0026	2.15×10^{-149}
8	rs6586884	20005847	*LPL*	T / C	0.1011	0.6842	-0.0889	0.0042	5.03×10^{-98}
19	rs438811	44913484	*AC011481.3*	C / T	0.1746	0.9292	0.0639	0.0034	9.43×10^{-81}
7	rs3812316	73606007	*MLXIPL*	C / G	0.0859	0.8849	-0.0728	0.0046	1.77×10^{-57}
8	rs2001945	125465736	*AC091114.1*	C / G	0.4368	0.7772	0.0351	0.0026	6.47×10^{-42}
15	rs2070895	58431740	*ALDH1A2, LIPC*	G / A	0.3827	0.463	0.0332	0.0026	1.77×10^{-36}
1	rs11207996	62589799	*DOCK7*	G / A	0.2097	0.1421	-0.0340	0.0031	2.75×10^{-27}
19	rs58542926	19268740	*AC138430.1, TM6SF2*	C / T	0.0662	0.6291	-0.0543	0.0051	1.44×10^{-26}
5	rs465002	56512648	*C5orf67*	T / C	0.4694	0.1535	-0.0272	0.0026	2.67×10^{-26}
2	rs35131127	20983042	*AC115619.1*	T / C	0.08703	0.9145	0.0450	0.0045	4.12×10^{-23}
11	rs174556	61813163	*FADS2, FADS1*	T / C	0.4214	0.009995	-0.0251	0.0026	7.73×10^{-22}
6	rs9264086	31233907	*AL662844.2*	G / A	0.4328	0.2469	0.0232	0.0026	2.79×10^{-19}
20	rs148753678	45907567	*PLTP*	CACA / C	0.0668	0.5506	0.0450	0.0052	2.90×10^{-18}
10	rs7084396	63116965	*RNU6-543P*	G / A	0.3843	0.6542	0.0225	0.0026	2.18×10^{-17}
6	rs1358980	43796814	*AL157371.2*	T / C	0.4486	0.7872	-0.0216	0.0026	4.15×10^{-17}

8	rs4921915	18414956	*NAT2*	G / A	0.4818	0.703	-0.0215	0.0026	4.54×10^{-17}
10	rs2263985	121143992	*RPL19P16*	T / C	0.4113	0.5373	-0.0215	0.0026	1.46×10^{-16}
7	rs34084575	130751643	*KLF14*	T / TA	0.3125	0.1542	-0.0210	0.0028	3.62×10^{-14}
6	rs198806	26133388	*HIST1H2AC*	G / A	0.1359	0.5134	0.0279	0.0037	9.54×10^{-14}
15	rs77648222	69890512	*DRAIC*	A / G	0.2346	0.6979	-0.0219	0.0030	3.89×10^{-13}
16	rs2925979	81501185	*CMIP*	C / T	0.4292	0.1979	0.0186	0.0026	6.03×10^{-13}
14	rs79823890	52045251	*NID2*	G / T	0.1186	0.734	-0.0284	0.0040	7.08×10^{-13}
9	rs78579422	109475873	*PTPN3*	C / T	0.2438	0.1536	-0.0213	0.0030	2.01×10^{-12}
5	rs6882076	156963286	*TIMD4*	C / T	0.2713	0.2033	-0.0201	0.0029	2.83×10^{-12}
19	rs1672991	35065755	*HPN, HPN-AS1*	G / A	0.1596	0.4968	-0.0238	0.0035	9.08×10^{-12}
6	rs12214804	34221089	*CYCSP55*	T / C	0.1084	0.442	-0.0282	0.0041	9.13×10^{-12}
3	rs1899951	12353341	*PPARG*	C / T	0.0397	0.0525	-0.0445	0.0065	1.07×10^{-11}
7	rs12534104	44180143	*GCK*	C / A	0.226	0.0646	-0.0212	0.0031	1.26×10^{-11}
4	rs10023050	87143279	*AFF1*	A / G	0.4101	0.559	-0.0172	0.0026	4.96×10^{-11}
8	rs11558471	117173494	*SLC30A8*	A / G	0.4676	0.7192	-0.0166	0.0026	9.39×10^{-11}
10	rs1972360	92636831	*KIF11*	T / C	0.3379	0.2404	0.0175	0.0027	1.08×10^{-10}
19	rs60748247	8435690	*MARCHF2*	C / CTACCACACCTGGCTAGT	0.0135	0.1339	-0.0688	0.0110	3.79×10^{-10}
9	rs2575876	104903458	*ABCA1*	G / A	0.2309	0.0246	-0.0188	0.0030	4.46×10^{-10}
4	rs11941723	17810905	*NCAPG*	A / G	0.1433	0.9353	0.0227	0.0037	6.13×10^{-10}

19	rs1423065	33359589	*AKR1B1P7*	C / T	0.1724	0.625	0.0208	0.0034	9.72×10^{-10}
22	rs7291040	46089093	*MIRLET7BHG*	T / C	0.1186	0.4999	-0.0239	0.0040	3.81×10^{-9}
12	rs10744776	109255001	*ACACB*	G / A	0.2507	0.1637	-0.0174	0.0030	5.51×10^{-9}
10	rs4919594	93063839	*CYP26C1, AL358613.3*	C / G	0.1764	0.7092	0.0196	0.0034	5.77×10^{-9}
2	rs1402837	168900844	*SPC25*	C / T	0.4042	0.05668	0.0151	0.0026	9.09×10^{-9}
11	rs7936331	13812845	*LINC02548*	G / A	0.488	0.7156	0.0146	0.0026	1.28×10^{-8}
4	rs10937924	3373171	*RGS12*	C / T	0.3734	0.1005	-0.0151	0.0027	2.13×10^{-8}
2	rs340515	44961231	*AC012354.1*	T / G	0.4369	0.5939	0.0144	0.0026	2.74×10^{-8}
11	rs163184	2825839	*KCNQ1*	T / G	0.4414	0.4078	0.0142	0.0026	3.10×10^{-8}
6	rs9368222	20686765	*CDKAL1*	C / A	0.3477	0.634	0.0149	0.0027	3.33×10^{-8}
12	rs117624317	110194076	*IFT81*	C / T	0.0979	0.603	0.0240	0.0043	3.59×10^{-8}
17	rs10775406	48120393	*SNX11*	G / A	0.2393	0.944	-0.0165	0.0030	3.74×10^{-8}
7	rs577611417	80651863	*CD36*	C / A	0.0197	1	-0.0506	0.0092	3.75×10^{-8}

CHR: chromosome; Ref/Alt: reference/alternate alleles; MAF: minor allele frequency; HWE: Hardy–Weinberg equilibrium; L95: Lower 95% CI, U95%: Upper 95% CI

P: adjusted for age, sex, BMI, current smoking and 10 principal components

Supplementary Table S7. TyG index and CKM stages according to TyG index -determining genotypes with genome-wide significance ($P < 5 \times 10^{-8}$) in Taiwan Biobank participants in TWB2 cohort

CHR	Lead variants	Position (GRCh38)	Gene	Ref/Alt	MAF	HWE	TyG index			CKM stages 0-advanced stages			CKM stages 0-advanced stages*		
							BETA	SE	*P*	BETA	SE	*P*	BETA	SE	*P*
11	rs7350481	116715567	*BUD13*	C / T	0.2374	0.315	0.1248	0.0030	$< 10^{-307}$	0.1754	0.0119	3.99×10^{-49}	-0.0181	0.0132	0.1714
2	rs6547692	27512105	*GCKR*	A / G	0.492	0.3354	0.0665	0.0026	2.15×10^{-149}	0.1002	0.0101	3.24×10^{-23}	0.0023	0.0111	0.8322
8	rs6586884	20005847	*LPL*	T / C	0.1011	0.6842	-0.0889	0.0042	5.03×10^{-98}	-0.0943	0.0167	1.53×10^{-8}	0.0561	0.0182	0.0020
19	rs438811	44913484	*AC011481.3*	C / T	0.1746	0.9292	0.0639	0.0034	9.43×10^{-81}	0.0459	0.0133	5.40×10^{-4}	-0.0371	0.0145	0.0105
7	rs3812316	73606007	*MLXIPL*	C / G	0.0859	0.8849	-0.0728	0.0046	1.77×10^{-57}	-0.0979	0.0180	5.05×10^{-8}	0.0122	0.0196	0.5333
8	rs2001945	125465736	*AC091114.1*	C / G	0.4368	0.7772	0.0351	0.0026	6.47×10^{-42}	0.0448	0.0102	1.10×10^{-5}	-0.0091	0.0111	0.4142
15	rs2070895	58431740	*ALDH1A2, LIPC*	G / A	0.3827	0.463	0.0332	0.0026	1.77×10^{-36}	0.0384	0.0103	0.0002	-0.0211	0.0113	0.0621
1	rs11207996	62589799	*DOCK7*	G / A	0.2097	0.1421	-0.0340	0.0031	2.75×10^{-27}	-0.0301	0.0124	0.0150	0.0113	0.0135	0.4038
19	rs58542926	19268740	*AC138430.1, TM6SF2*	C / T	0.0662	0.6291	-0.0543	0.0051	1.44×10^{-26}	-0.0800	0.0200	6.30×10^{-5}	-0.0030	0.0217	0.8893
5	rs465002	56512648	*C5orf67*	T / C	0.4694	0.1535	-0.0272	0.0026	2.67×10^{-26}	-0.0703	0.0101	3.49×10^{-12}	-0.0213	0.0110	0.0531
2	rs35131127	20983042	*AC115619.1*	T / C	0.08703	0.9145	0.0450	0.0045	4.12×10^{-23}	0.0656	0.0179	0.0003	0.0038	0.0196	0.8469
11	rs174556	61813163	*FADS2, FADS1*	T / C	0.4214	0.009995	-0.0251	0.0026	7.73×10^{-22}	-0.0232	0.0103	0.0237	0.0127	0.0112	0.2565
6	rs9264086	31233907	*AL662844.2*	G / A	0.4328	0.2469	0.0232	0.0026	2.79×10^{-19}	0.0178	0.0102	0.0797	-0.0225	0.0111	0.0423
20	rs148753678	45907567	*PLTP*	CACA / C	0.0668	0.5506	0.0450	0.0052	2.90×10^{-18}	0.0154	0.0203	0.4476	-0.0606	0.0222	0.0063
10	rs7084396	63116965	*RNU6-543P*	G / A	0.3843	0.6542	0.0225	0.0026	2.18×10^{-17}	0.0484	0.0104	4.00×10^{-6}	0.0140	0.0114	0.2178

6	rs1358980	43796814	*AL157371.2*	T / C	0.4486	0.7872	-0.0216	0.0026	4.15×10^{-17}	-0.0356	0.0101	4.31×10^{-4}	-0.0011	0.0110	0.9237
8	rs4921915	18414956	*NAT2*	G / A	0.4818	0.703	-0.0215	0.0026	4.54×10^{-17}	-0.0205	0.0101	0.0427	0.0128	0.0110	0.2452
10	rs2263985	121143992	*RPL19P16*	T / C	0.4113	0.5373	-0.0215	0.0026	1.46×10^{-16}	-0.0849	0.0102	1.09×10^{-16}	-0.0515	0.0112	4.00×10^{-6}
7	rs34084575	130751643	*KLF14*	T / TA	0.3125	0.1542	-0.0210	0.0028	3.62×10^{-14}	-0.0812	0.0109	1.18×10^{-13}	-0.0395	0.0119	9.33×10^{-4}
6	rs198806	26133388	*HIST1H2AC*	G / A	0.1359	0.5134	0.0279	0.0037	9.54×10^{-14}	0.0536	0.0147	0.0003	0.0188	0.0161	0.2429
15	rs77648222	69890512	*DRAIC*	A / G	0.2346	0.6979	-0.0219	0.0030	3.89×10^{-13}	-0.0537	0.0119	6.00×10^{-6}	-0.0177	0.0130	0.1726
16	rs2925979	81501185	*CMIP*	C / T	0.4292	0.1979	0.0186	0.0026	6.03×10^{-13}	0.0429	0.0102	2.40×10^{-5}	0.0055	0.0111	0.6226
14	rs79823890	52045251	*NID2*	G / T	0.1186	0.734	-0.0284	0.0040	7.08×10^{-13}	-0.1004	0.0156	1.19×10^{-10}	-0.0522	0.0170	0.0022
9	rs78579422	109475873	*PTPN3*	C / T	0.2438	0.1536	-0.0213	0.0030	2.01×10^{-12}	-0.0436	0.0120	0.0003	-0.0119	0.0130	0.3620
5	rs6882076	156963286	*TIMD4*	C / T	0.2713	0.2033	-0.0201	0.0029	2.83×10^{-12}	-0.0235	0.0114	0.0388	0.0077	0.0124	0.5329
19	rs1672991	35065755	*HPN, HPN-AS1*	G / A	0.1596	0.4968	-0.0238	0.0035	9.08×10^{-12}	-0.0241	0.0138	0.0803	0.0164	0.0150	0.2730
6	rs12214804	34221089	*CYCSP55*	T / C	0.1084	0.442	-0.0282	0.0041	9.13×10^{-12}	-0.0741	0.0163	6.00×10^{-6}	-0.0375	0.0178	0.0349
3	rs1899951	12353341	*PPARG*	C / T	0.0397	0.0525	-0.0445	0.0065	1.07×10^{-11}	-0.1132	0.0258	1.20×10^{-5}	-0.0282	0.0280	0.3143
7	rs12534104	44180143	*GCK*	C / A	0.226	0.0646	-0.0212	0.0031	1.26×10^{-11}	-0.0530	0.0123	1.60×10^{-5}	-0.0336	0.0134	0.0121
4	rs10023050	87143279	*AFF1*	A / G	0.4101	0.559	-0.0172	0.0026	4.96×10^{-11}	-0.0140	0.0103	0.1746	0.0119	0.0112	0.2895
8	rs11558471	117173494	*SLC30A8*	A / G	0.4676	0.7192	-0.0166	0.0026	9.39×10^{-11}	-0.0569	0.0101	1.69×10^{-8}	-0.0229	0.0110	0.0378
10	rs1972360	92636831	*KIF11*	T / C	0.3379	0.2404	0.0175	0.0027	1.08×10^{-10}	0.0509	0.0107	2.00×10^{-6}	0.0156	0.0117	0.1830
19	rs60748247	8435690	*MARCHF2*	C / CTACCACACCTGGCTAGT	0.0135	0.1339	-0.0688	0.0110	3.79×10^{-10}	-0.1583	0.0431	0.0002	-0.0675	0.0466	0.1474
9	rs2575876	104903458	*ABCA1*	G / A	0.2309	0.0246	-0.0188	0.0030	4.46×10^{-10}	-0.0202	0.0119	0.0894	0.0064	0.0130	0.6203

4	rs11941723	17810905	*NCAPG*	A / G	0.1433	0.9353	0.0227	0.0037	6.13×10^{-10}	0.0809	0.0145	2.51×10^{-8}	0.0404	0.0159	0.0109
19	rs1423065	33359589	*AKR1B1P7*	C / T	0.1724	0.625	0.0208	0.0034	9.72×10^{-10}	0.0381	0.0134	0.0044	0.0037	0.0146	0.8012
22	rs7291040	46089093	*MIRLET7BHG*	T / C	0.1186	0.4999	-0.0239	0.0040	3.81×10^{-9}	-0.0317	0.0160	0.0468	0.0083	0.0174	0.6333
12	rs10744776	109255001	*ACACB*	G / A	0.2507	0.1637	-0.0174	0.0030	5.51×10^{-9}	-0.0380	0.0117	0.0012	-0.0145	0.0128	0.2568
10	rs4919594	93063839	*CYP26C1, AL358613.3*	C / G	0.1764	0.7092	0.0196	0.0034	5.77×10^{-9}	0.0198	0.0133	0.1359	-0.0086	0.0145	0.5537
2	rs1402837	168900844	*SPC25*	C / T	0.4042	0.05668	0.0151	0.0026	9.09×10^{-9}	0.0316	0.0103	0.0022	0.0124	0.0113	0.2706
11	rs7936331	13812845	*LINC02548*	G / A	0.488	0.7156	0.0146	0.0026	1.28×10^{-8}	0.0159	0.0101	0.1156	-0.0061	0.0110	0.5788
4	rs10937924	3373171	*RGS12*	C / T	0.3734	0.1005	-0.0151	0.0027	2.13×10^{-8}	-0.0170	0.0106	0.1079	0.0015	0.0115	0.8952
2	rs340515	44961231	*AC012354.1*	T / G	0.4369	0.5939	0.0144	0.0026	2.74×10^{-8}	0.0338	0.0102	0.0009	0.0216	0.0112	0.0533
11	rs163184	2825839	*KCNQ1*	T / G	0.4414	0.4078	0.0142	0.0026	3.10×10^{-8}	0.0512	0.0101	4.19×10^{-7}	0.0117	0.0111	0.2907
6	rs9368222	20686765	*CDKAL1*	C / A	0.3477	0.634	0.0149	0.0027	3.33×10^{-8}	0.0659	0.0106	4.52×10^{-10}	0.0263	0.0116	0.0233
12	rs117624317	110194076	*IFT81*	C / T	0.0979	0.603	0.0240	0.0043	3.59×10^{-8}	0.0776	0.0172	6.00×10^{-6}	0.0482	0.0188	0.0102
17	rs10775406	48120393	*SNX11*	G / A	0.2393	0.944	-0.0165	0.0030	3.74×10^{-8}	-0.0446	0.0118	0.0002	-0.0135	0.0129	0.2954
7	rs577611417	80651863	*CD36*	C / A	0.0197	1	-0.0506	0.0092	3.75×10^{-8}	-0.0031	0.0363	0.9310	0.1204	0.0395	0.0023

CHR: chromosome; Ref/Alt: reference/alternate alleles; MAF: minor allele frequency; HWE: Hardy–Weinberg equilibrium

P: adjusted for age, sex, BMI, current smoking and 10 principal components, **P*: adjusted for age, sex, BMI, current smoking, 10 principal components and TyG index

Red-highlighted variants were excluded from the instrumental variable set because they showed evidence of direct association with CKM stages outcomes ($P < 0.01$) independent of the TyG index, to minimize potential horizontal pleiotropy.

Supplementary Table S8. Summary of genetic association estimates used for Mendelian randomization analyses of the TyG index and CKM stages (0 to advanced) in non-overlapping Taiwan Biobank subsets.

T_B	T_A-T_B (TWB2, N=75,402)		G_A-T_A (TWB2, N=75,402)		G_A-T_B (TWB1, N=20,757)		IV_A-T_B (TWB1, N=20,757)			IV_A-T_B+T_A (TWB1, N=20,757)	
CKM stages	Beta (SE)	*P*‡	Beta (SE)	*P*	Beta (SE)	*P*	Beta (SE)	*P*	*P*§	Beta (SE)	*P*
Stage 0-advanced stages*	1.7754 (0.0172)	$< 10^{-307}$	1.0074 (0.0159)	$< 10^{-307}$	1.4269 (0.1225)	2.36×10^{-31}	1.4170 (0.1217)	2.36×10^{-31}	1.28×10^{-22}	0.0270 (0.1290)	0.8342
Stage 0-advanced stages †	1.7516 (0.0166)	$< 10^{-307}$	1.0087 (0.0160)	$< 10^{-307}$	1.3955) (0.1204	4.38×10^{-31}	1.3858 (0.1195)	4.38×10^{-31}	3.23×10^{-21}	0.0214 (0.1264)	0.8657
Stages ≥2 vs. stages 0–1*	2.2225 (0.0223)	$< 10^{-307}$	1.0074 (0.0159)	$< 10^{-307}$	1.8425 (0.1375)	6.45×10^{-41}	1.8296 (0.1366)	6.45×10^{-41}	6.89×10^{-32}	0.2070 (0.1523)	0.1741
Stages ≥2 vs. stages 0–1†	2.2362 (0.0220)	$< 10^{-307}$	1.0087 (0.0160)	$< 10^{-307}$	1.8415 (0.1362)	1.12×10^{-41}	1.8287 (0.1352)	1.12×10^{-41}	5.82×10^{-31}	0.2215 (0.1510)	0.1424
Stages ≥1 vs. stage 0*	1.3218) (0.0314)	$< 10^{-307}$	1.0074 (0.0159)	$< 10^{-307}$	0.7688 (0.2085)	2.28×10^{-4}	0.7635 (0.2071)	2.28×10^{-4}	0.0055	-0.1424 (0.2174	0.5126
Stages ≥1 vs. stage 0†	1.3432 (0.0311)	$< 10^{-307}$	1.0087 (0.0160)	$< 10^{-307}$	0.8043 (0.2076)	1.08×10^{-4}	0.7987 (0.2061)	1.08×10^{-4}	0.0051	-0.1382) (0.2165)	0.5232

Exclusion of lead variants when the association with outcome showed *P* <0.01 after exposure adjustment in each MR analysis

* Exclusion of participants with a history of hyperlipidemia and diabetes mellitus

† Exclusion of participants with a history of hyperlipidemia

P: Adjusted for age, sex, smoking, body mass index and 10 *principal components*

P‡*:* Adjusted for age, sex, smoking, body mass index

P§: For multivariate analysis: *: Further adjusted for alcohol drinking, physical activity, HDL-C, LDL-C, hypertension, chronic kidney disease, education status; †: Further adjusted for alcohol drinking, physical activity, HDL-C, LDL-C, hypertension, diabetes mellitus, chronic kidney disease, education status.

Abbreviations: IV_A, instrumental variables for G_A; T_A: TyG index; G_A: wGRS_TyG index _TWB2_42SNPs; GWAS, genome-wide association study; Beta: beta-coefficient; SE: standard error; wGRS, weighted genetic risk score; HDL-C: high-density lipoprotein cholesterol level; LDL-C: low-density lipoprotein cholesterol level.
